# Trends in metabolic dysfunction-associated steatotic liver disease by household income, 2007–2022: A national representative study in South Korea

**DOI:** 10.1097/MD.0000000000045296

**Published:** 2025-10-24

**Authors:** Yerin Cho, Hyunjee Kim, Jinyoung Jeong, Jiyeon Oh, Jaeyu Park, Jaewon Kim, Jiyoung Hwang, Dong Keon Yon

**Affiliations:** a Center for Digital Health, Medical Science Research Institute, Kyung Hee University Medical Center, Kyung Hee University College of Medicine, Seoul, South Korea; b Department of Medicine, Kyung Hee University College of Medicine, Seoul, South Korea; c Department of Precision Medicine, Kyung Hee University College of Medicine, Seoul, South Korea; d Department of Regulatory Science, Kyung Hee University, Seoul, South Korea.

**Keywords:** household income level, metabolic dysfunction-associated steatotic liver disease, prevalence, South Korea, trend

## Abstract

Metabolic dysfunction-associated steatotic liver disease (MASLD) could lead to liver-related life-threatening conditions. However, relatively little research has been conducted on trends in MASLD based on household income. This study aimed to examine trends in MASLD prevalence stratified by household income and identify socioeconomic factors associated with a risk of MASLD. Data from the Korea National Health and Nutrition Examination Survey from 2007 to 2022 were analyzed to examine MASLD prevalence among adults in South Korea, stratified by household income levels. Household income was categorized into quartiles, and covariates including sex, age, region of residence, education level, stress level, smoking status, and drinking status were assessed. Weighted prevalence estimates were calculated to assess trends in MASLD across the study period. In addition, logistic regression models were applied to estimate adjusted odds ratios with 95% confidence intervals (CIs), comparing pre-pandemic and pandemic periods as well as across income quartiles stratified by sex, age, and other covariates. A total of 70,276 individuals aged ≥19 years (male: 29,169 [41.51%]) from 2007 to 2022 were included in the analysis. MASLD prevalence increased over the study period across all household income levels. Particularly, in the medium-high and high household income levels, prevalence increased from 16.30% (95% CI, 14.83–17.76) in 2007 to 27.25% (95% CI, 24.46–30.04) in 2022 and from 15.15% (95% CI, 13.71–16.58) in 2007 to 25.44% (95% CI, 22.01–28.86) in 2022, respectively. The prevalence of MASLD exhibited a sex-specific trend, being higher among low-income females while tending to be higher among high-income males. Males, middle-aged individuals, high-stress levels, and smoking status were identified as risk factors for MASLD. This is the first study to analyze the trends of MASLD stratified by household income level in South Korea. These findings offer insights that may facilitate the identification of priority populations and guide the development of equity-focused public health strategies.

## 1. Introduction

Metabolic dysfunction-associated steatotic liver disease (MASLD), previously known as nonalcoholic fatty liver disease, is one of the most common chronic liver diseases globally.^[1]^ MASLD currently affects over 38% of the global population,^[2]^ and in South Korea, it is prevalent among more than one-third of the adult population.^[3]^ Furthermore, MASLD is associated with both liver-specific pathologies and a wide range of life-threatening extrahepatic complications, such as cirrhosis, hepatocellular carcinoma, and cardiovascular disease.^[4]^ These complications significantly contribute to the burden of morbidity and disability among affected individuals, highlighting the necessity for further research.^[5]^

MASLD is a significant disease closely linked to cardiometabolic risk factors such as obesity, which are influenced by socioeconomic conditions, particularly household income.^[6–8]^ Consequently, examining the prevalence of MASLD by income level is essential for understanding its socioeconomic determinants.^[9]^ Previous studies have reported that the prevalence of MASLD is lower among high-income groups, suggesting that socioeconomic disparities influence the disease burden.^[6]^ However, in South Korea, research examining temporal trends in MASLD prevalence by household income remains notably limited, despite the condition’s growing clinical and public health relevance. A comprehensive understanding of these patterns and their associated factors is essential for addressing future disease burden and formulating effective public health strategies.

In this study, we conducted a repeated cross-sectional analysis of 16 years of nationally representative data from the Korea National Health and Nutrition Examination Survey (KNHANES) to assess trends in MASLD prevalence by household income. By leveraging independent annual survey samples, we evaluated population-level patterns and examined income-related risk factors. To our knowledge, this is the first nationally representative study in South Korea to investigate long-term trends in MASLD prevalence across income levels. These findings provide insights that may help identify priority populations and inform future equity-focused public health strategies.

## 2. Materials and methods

### 2.1. Study population and data collection

This study utilized KNHANES data from 2007 to 2022. The KNHANES is an annual, nationally representative survey conducted by the Korea Disease Control and Prevention Agency. Participants were randomly selected from 600 districts across South Korea to form a representative sample of the population.^[10]^ Individuals who completed a health examination, health interview, and nutritional survey were included in the study. Participants aged 18 years or younger, pregnant individuals, those with missing data, or those with hepatitis B or C virus infection, a history of liver cancer, or liver cirrhosis were excluded to ensure the accuracy of the analyses.^[11]^

A total of 70,276 participants were included as a nationally representative sample to assess the prevalence of MASLD stratified by household income levels. Data were collected for each participant on variables including sex, age, household income levels, region of residence, education level, stress level, alcohol consumption, smoking status, body mass index classification, dietary interview, health examination, and health survey results. The survey was conducted over 16 years, with the following number of participants in each year group: 2007 to 2009: 13,341; 2010 to 2012: 14,023; 2013 to 2015: 11,881; 2016 to 2019: 18,735; 2020: 3881; 2021: 4031, and 2022: 4384.

The research protocol was approved by the Institutional Review Boards of the Korea Disease Control and Prevention Agency (2007-02CON-04-P, 2008-04EXP-01-C, 2009-01CON-03-2C, 2010-02CON-21-C, 2011-02CON-06-C, 2012-01EXP-01-2C, 2013-07CON-03-4C, 2013-12EXP-035C, 2018-01-03-P-A, 2018-01-03-C-A, 2018-01-03-2C-A, 2018-01-03-5C-A, 2018-01-03-4C-A, 2022-11-16-R-A). Written informed consent was obtained from all participants prior to data collection. All procedures followed the ethical standards of the relevant national and institutional review boards and adhered to the principles of the Declaration of Helsinki, as revised in 2008. KNHANES provides publicly accessible data that may be used for a broad range of epidemiological research.

### 2.2. Definition of MASLD with household income level

This study aimed to examine the trends in the prevalence of MASLD among Korean adults by household income level. MASLD was defined as steatotic liver disease (SLD) with the presence of at least 1 of 5 cardiometabolic risk factors, which are components of metabolic syndrome: body mass index ≥ 23 kg/m^2^ or waist circumference ≥ 90 cm for males and ≥ 85 cm for females; fasting serum glucose ≥ 100 mg/dL or HbA1c ≥ 5.7% or undergoing specific drug treatment; blood pressure ≥ 130/85 mm Hg or antihypertensive drug treatment; triglycerides ≥ 150 mg/dL or lipid-lowering treatment; and plasma high-density lipoprotein cholesterol <40 mg/dL for male and <50 mg/dL for female or specific drug treatment, based on the presence of hepatic steatosis.^[11]^ MASLD was defined as an alcohol intake of <210 g/wk for males and <140 g/wk for females.^[12]^ Alcohol consumption was calculated using 24-hour dietary recall data and the food code table provided by the KNHANES nutrition survey.

Hepatic steatosis was assessed using the hepatic steatosis index (HSI), a noninvasive screening tool developed specifically for the Korean population. HSI is widely utilized in epidemiological studies due to its reasonable accuracy in evaluating hepatic steatosis.^[13]^ HSI was calculated based on the alanine aminotransferase/aspartate aminotransferase ratio, diabetes status, and sex, according to the following equation: HSI = (8 × alanine aminotransferase/aspartate aminotransferase ratio + 2 [if diabetes mellitus] + 2 [if female]).^[14]^ Individuals with an HSI value of ≥36 were classified as having hepatic steatosis.^[14]^ Participants were stratified into 4 groups (low, low-medium, medium-high, and high) based on equivalized household income, which was calculated by dividing the total household income by the square root of the household size. To address income variations across survey years, the quartiles of the annual monthly average equivalized household income were used as the classification criteria.^[15,16]^

### 2.3. Covariates

The covariates considered in this study included sex, age (19–29, 30–39, 40–49, 50–59, and ≥60 years), region of residence (urban and rural), education level (elementary school or lower, middle school, high school, and college or higher education), household income (low, low-medium, medium-high, and high quartiles), level of stress (high and low), smoking status (current, ex-smokers, and nonsmokers), and drinking status (nondrinker, 1–5 times/mo, and <6 times/mo).^[17]^ Information on physical measurements, including height, weight, and waist circumference, was obtained through direct measurement, while other variables were collected through self-reported data obtained from the health examination and health surveys.

### 2.4. Statistical analyses

Stratified analyses were performed based on household income levels, categorized into quartiles. For each income level, covariates including sex, age, region of residence, education level, stress level, smoking status, and drinking status were assessed. Weighted prevalence estimates were calculated to assess trends in MASLD across the study period. To examine trends in MASLD prevalence, multivariable weighted logistic regression models were applied with survey year treated both as a continuous variable to evaluate linear trends and as a categorical variable grouped into 4 periods (2007–2010, 2011–2014, 2015–2018, and 2019–2022) to assess period-specific differences.^[18]^ The year 2020, marking the onset of the COVID-19 pandemic in South Korea,^[19,20]^ was designated as a point to compare pre-pandemic and pandemic trends using β coefficients derived from linear regression models with 95% confidence intervals (CIs). Additionally, β differences (β_diff_) was calculated to examine the change in prevalence before and during the pandemic. Adjusted odds ratios (aORs) and corresponding 95% CIs were estimated using multivariable weighted logistic regression models, adjusting for the covariates. The ratio of aORs was also calculated to assess changes in MASLD prevalence before and during the pandemic. Additionally, with the highest household income quartile designated as the reference group, aORs and 95% CIs were estimated for the remaining quartiles, stratified by sex, age, and other covariates, to evaluate the association between household income and MASLD prevalence. The statistical significance of β coefficients, β_diff_, and aORs was evaluated using 2-sided *P*-values, with values <.05 considered statistically significant. All statistical analyses were performed using SAS software (version 9.4; SAS Institute, Cary).

## 3. Results

### 3.1. Study population characteristics

This analysis utilized data from the KNHANES from 2007 to 2022. Of the 1,26,446 participants in the KNHANES dataset, the following exclusions were applied: individuals aged 18 years or younger, pregnant women, those with missing data, individuals with hepatitis B or C virus infections, and those with a history of liver cancer or liver cirrhosis. Therefore 70,276 participants were included in the final sample for this study (Fig. S1, Supplemental Digital Content, https://links.lww.com/MD/Q375).

The database included the demographic characteristics of the participants, summarized as follows: sex (29,169 males [41.51%] and 41,107 females [58.49%]) and age groups (19–29 years: 7836 [11.15%]; 30–39 years: 11,796 [16.79%]; 40–49 years: 12,943 [18.42%]; 50–59 years: 13,239 [18.84%]; and ≥60 years: 24,462 [34.81%]; Table 1).

**Table 1:** Baseline characteristics of Korean adults based on the data from the KNHANES, 2007–2022 (n = 70,276).

Characteristic	Total	2007–2009	2010–2012	2013–2015	2016–2019	2020	2021	2022
Overall, n	70,276	13,341	14,023	11,881	18,735	3881	4031	4384
Crude rate, n (%)								
Sex, n (%)								
Male	29,169 (41.51)	5343 (50.00)	5676 (50.12)	4908 (49.94)	7918 (49.94)	1680 (50.05)	1730 (49.99)	1914 (50.26)
Female	41,107 (58.49)	7998 (50.00)	8347 (49.88)	6973 (50.06)	10,817 (50.06)	2201 (49.95)	2301 (50.01)	2470 (49.74)
Age (yr), n (%)								
19–29	7836 (11.15)	1527 (19.61)	1432 (18.62)	1318 (18.31)	2061 (17.37)	519 (17.62)	461 (17.14)	518 (16.67)
30–39	11,796 (16.79)	2721 (22.80)	2556 (21.15)	1973 (19.57)	2946 (17.97)	536 (17.58)	471 (16.85)	593 (16.57)
40–49	12,943 (18.42)	2633 (22.72)	2492 (22.24)	2225 (21.69)	3501 (20.49)	683 (20.06)	678 (19.31)	731 (18.83)
50–59	13,239 (18.84)	2293 (16.65)	2767 (18.59)	2393 (20.09)	3527 (20.17)	724 (20.05)	739 (19.44)	796 (20.01)
≥60	24,462 (34.81)	4167 (18.22)	4776 (19.39)	3972 (20.34)	6700 (24.01)	1419 (24.69)	1682 (27.26)	1746 (27.92)
Region of residence, n (%)								
Urban	55,191 (78.53)	9665 (81.05)	6291 (45.00)	9627 (83.67)	15,211 (86.37)	3143 (87.00)	3118 (84.76)	3424 (84.60)
Rural	15,085 (21.47)	3676 (18.95)	7732 (55.00)	2254 (16.33)	3524 (13.63)	738 (13.00)	913 (15.24)	960 (15.40)
Level of education, n (%)								
Elementary school or lower education	12,047 (17.14)	1080 (4.32)	3372 (16.35)	2367 (13.07)	3323 (11.80)	554 (9.33)	682 (10.05)	669 (9.48)
Middle school	10,495 (14.93)	4250 (23.80)	1675 (10.11)	1369 (9.14)	2000 (8.24)	398 (7.55)	418 (7.19)	385 (6.62)
High school	19,640 (27.95)	3917 (32.20)	3959 (30.46)	3415 (29.35)	4994 (26.71)	1067 (27.27)	1099 (27.77)	1189 (26.61)
College or higher education	28,094 (39.98)	4094 (39.68)	5017 (43.09)	4730 (48.45)	8418 (53.25)	1862 (55.85)	1832 (54.98)	2141 (57.30)
Household income, n (%)								
Low	13,665 (19.44)	2872 (15.68)	2748 (15.31)	2174 (13.74)	3572 (14.75)	655 (13.18)	796 (13.54)	848 (14.85)
Low-medium	17,537 (24.95)	3316 (24.36)	3619 (27.24)	3046 (24.86)	4619 (23.51)	918 (21.76)	958 (22.25)	1061 (22.00)
Medium-high	19,139 (27.23)	3569 (29.54)	3838 (29.72)	3272 (29.90)	5033 (28.83)	1110 (29.88)	1108 (30.66)	1209 (30.07)
High	19,935 (28.37)	3584 (30.43)	3818 (27.74)	3389 (31.50)	5511 (32.91)	1198 (35.18)	1169 (33.54)	1266 (33.08)
Level of stress, n (%)								
High-stress level	18,229 (25.94)	3733 (29.14)	3600 (27.26)	2850 (25.96)	4924 (27.77)	1056 (28.83)	1003 (26.16)	1063 (26.02)
Low-stress level	52,047 (74.06)	9608 (70.86)	10,423 (72.74)	9031 (74.04)	13,811 (72.23)	2825 (71.17)	3028 (73.84)	3321 (73.98)
Smoking status, n (%)								
Smoker	12,446 (17.71)	2704 (26.42)	2600 (25.50)	2136 (22.84)	3112 (20.29)	634 (18.80)	594 (17.92)	666 (17.09)
Ex-smoker	14,853 (21.14)	2574 (20.46)	2895 (20.91)	2376 (20.50)	4110 (22.61)	875 (24.48)	947 (24.58)	1076 (26.46)
Nonsmoker	42,977 (61.15)	8063 (53.12)	8528 (53.59)	7369 (56.66)	11,513 (57.10)	2372 (56.72)	2490 (57.50)	2642 (56.46)
Drinking frequency status, n (%)								
Nondrinker	20,338 (28.94)	3990 (23.67)	4046 (22.47)	3387 (23.11)	5158 (22.47)	1167 (25.12)	1344 (27.25)	1246 (24.25)
1–5 times/mo	35,433 (50.42)	6589 (53.16)	7164 (54.41)	6037 (53.94)	9526 (54.06)	1933 (53.23)	1958 (52.62)	2226 (53.63)
<6 times/mo	14,505 (20.64)	2762 (23.18)	2813 (23.13)	2457 (22.95)	4051 (23.47)	781 (21.65)	729 (20.12)	912 (22.12)
MASLD propotion, n (%)								
No	56,784 (80.80)	11,038 (83.09)	11,822 (83.95)	9706 (81.85)	14,813 (78.69)	2949 (75.54)	3131 (76.39)	3325 (74.45)
Yes	13,492 (19.20)	2303 (16.91)	2201 (16.05)	2175 (18.15)	3922 (21.31)	932 (24.46)	900 (23.61)	1059 (25.55)
Weighted rate (95% CI)								
Sex, weighted % (95% CI)								
Male	50.02 (49.63–50.41)	50.00 (49.10–50.90)	50.12 (49.23–51.02)	49.94 (49.02–50.86)	49.94 (49.22–50.66)	50.05 (48.60–51.50)	49.99 (48.17–51.82)	50.26 (48.75–51.77)
Female	49.98 (49.59–50.37)	50.00 (49.10–50.90)	49.88 (48.98–50.77)	50.06 (49.14–50.98)	50.06 (49.34–50.78)	49.95 (48.50–51.40)	50.01 (48.18–51.83)	49.74 (48.23–51.25)
Age (yr), weighted % (95% CI)								
19–29	18.02 (17.52–18.53)	19.61 (18.31–20.90)	18.62 (17.41–19.84)	18.31 (17.14–19.48)	17.37 (16.43–18.32)	17.62 (15.85–19.40)	17.14 (15.02–19.27)	16.67 (14.76–18.57)
30–39	19.29 (18.76–19.82)	22.80 (21.37–24.24)	21.15 (19.96–22.34)	19.57 (18.35–20.78)	17.97 (16.98–18.95)	17.58 (15.38–19.78)	16.85 (14.90–18.81)	16.57 (14.39–18.76)
40–49	21.10 (20.60–21.59)	22.72 (21.47–23.97)	22.24 (21.06–23.41)	21.69 (20.59–22.79)	20.49 (19.60–21.37)	20.06 (18.03–22.09)	19.31 (17.27–21.35)	18.83 (16.70–20.95)
50–59	19.32 (18.90–19.74)	16.65 (15.71–17.60)	18.59 (17.68–19.50)	20.09 (19.09–21.09)	20.17 (19.36–20.97)	20.05 (18.25–21.86)	19.44 (17.65–21.23)	20.01 (18.42–21.61)
≥60	22.27 (21.75–22.79)	18.22 (17.15–19.28)	19.39 (18.35–20.44)	20.34 (19.27–21.42)	24.01 (22.96–25.06)	24.69 (22.16–27.21)	27.26 (24.92–29.59)	27.92 (25.52–30.32)
Region of residence, weighted % (95% CI)								
Urban	83.79 (82.52–85.05)	81.05 (77.69–84.41)	80.17 (76.92–83.43)	83.67 (80.81–86.52)	86.37 (84.12–88.61)	87.00 (82.06–91.94)	84.76 (79.92–89.59)	84.60 (79.37–89.83)
Rural	16.21 (14.95–17.48)	18.95 (15.59–22.31)	19.83 (16.57–23.08)	16.33 (13.48–19.19)	13.63 (11.39–15.88)	13.00 (8.06–17.94)	15.24 (10.41–20.08)	15.40 (10.17–20.63)
Level of education, weighted % (95% CI)								
Elementary school or lower education	11.47 (11.10–11.84)	4.32 (3.87–4.78)	16.35 (15.36–17.33)	13.07 (12.17–13.97)	11.80 (11.08–12.51)	9.33 (7.89–10.77)	10.05 (8.58–11.52)	9.48 (8.21–10.74)
Middle school	10.54 (10.23–10.86)	23.80 (22.53–25.06)	10.11 (9.45–10.76)	9.14 (8.48–9.79)	8.24 (7.73–8.76)	7.55 (6.44–8.66)	7.19 (6.22–8.17)	6.62 (5.67–7.56)
High school	28.73 (28.22–29.24)	32.20 (30.92–33.48)	30.46 (29.28–31.64)	29.35 (28.13–30.56)	26.71 (25.74–27.67)	27.27 (25.13–29.41)	27.77 (25.85–29.70)	26.61 (24.72–28.50)
College or higher education	49.26 (48.51–50.01)	39.68 (37.91–41.45)	43.09 (41.48–44.69)	48.45 (46.83–50.07)	53.25 (51.75–54.75)	55.85 (52.44–59.25)	54.98 (51.98–57.99)	57.30 (54.73–59.86)
Household income level, weighted % (95% CI)								
Low	14.61 (14.14–15.08)	15.68 (14.53–16.83)	15.31 (14.23–16.39)	13.74 (12.68–14.80)	14.75 (13.82–15.68)	13.18 (11.17–15.18)	13.54 (11.60–15.49)	14.85 (13.40–16.30)
Low-medium	24.26 (23.68–24.85)	24.36 (22.89–25.83)	27.24 (25.80–28.67)	24.86 (23.40–26.32)	23.51 (22.47–24.54)	21.76 (19.44–24.09)	22.25 (20.13–24.38)	22.00 (19.98–24.03)
Medium-high	29.58 (29.00–30.17)	29.54 (28.05–31.02)	29.72 (28.42–31.01)	29.90 (28.40–31.40)	28.83 (27.77–29.89)	29.88 (27.61–32.15)	30.66 (28.26–33.07)	30.07 (27.76–32.39)
High	31.55 (30.75–32.34)	30.43 (28.48–32.38)	27.74 (26.22–29.26)	31.50 (29.59–33.41)	32.91 (31.39–34.43)	35.18 (31.65–38.70)	33.54 (29.74–37.33)	33.08 (30.12–36.04)
Level of stress, weighted % (95% CI)								
High-stress level	27.34 (26.90–27.78)	29.14 (28.14–30.14)	27.26 (26.28–28.23)	25.96 (24.94–26.99)	27.77 (26.97–28.57)	28.83 (26.51–31.16)	26.16 (24.51–27.80)	26.02 (24.13–27.91)
Low-stress level	72.66 (72.22–73.10)	70.86 (69.86–71.86)	72.74 (71.77–73.72)	74.04 (73.01–75.06)	72.23 (71.43–73.03)	71.17 (68.84–73.49)	73.84 (72.20–75.49)	73.98 (72.09–75.87)
Smoking status, weighted % (95% CI)								
Smoker	22.04 (21.60–22.48)	26.42 (25.35–27.48)	25.50 (24.49–26.52)	22.84 (21.79–23.88)	20.29 (19.48–21.09)	18.80 (17.18–20.42)	17.92 (16.34–19.51)	17.09 (15.37–18.81)
Ex-smoker	22.17 (21.81–22.54)	20.46 (19.62–21.30)	20.91 (20.04–21.78)	20.50 (19.66–21.34)	22.61 (21.94–23.28)	24.48 (22.99–25.98)	24.58 (23.06–26.10)	26.46 (24.91–28.01)
Nonsmoker	55.78 (55.34–56.23)	53.12 (52.05–54.19)	53.59 (52.60–54.57)	56.66 (55.66–57.67)	57.10 (56.25–57.95)	56.72 (54.87–58.56)	57.50 (55.60–59.40)	56.46 (54.68–58.24)
Drinking frequency status, weighted % (95% CI)								
Nondrinker	23.40 (22.97–23.83)	23.67 (22.64–24.70)	22.47 (21.50–23.44)	23.11 (22.13–24.09)	22.47 (21.69–23.25)	25.12 (23.10–27.14)	27.25 (25.45–29.06)	24.25 (22.56–25.93)
1–5 times/mo	53.79 (53.30–54.29)	53.16 (51.95–54.36)	54.41 (53.19–55.62)	53.94 (52.77–55.11)	54.06 (53.16–54.96)	53.23 (51.03–55.42)	52.62 (50.57–54.67)	53.63 (51.77–55.50)
<6 times/mo	22.81 (22.40–23.21)	23.18 (22.27–24.08)	23.13 (22.16–24.09)	22.95 (21.97–23.93)	23.47 (22.71–24.23)	21.65 (20.08–23.23)	20.12 (18.49–21.76)	22.12 (20.50–23.74)
MASLD, n (%)								
No	80.16 (79.75–80.57)	83.09 (82.25–83.94)	83.95 (83.06–84.84)	81.85 (81.01–82.69)	78.69 (77.92–79.47)	75.54 (73.81–77.27)	76.39 (74.55–78.23)	74.45 (72.59–76.32)
Yes	19.84 (19.43–20.25)	16.91 (16.06–17.75)	16.05 (15.16–16.94)	18.15 (17.31–18.99)	21.31 (20.53–22.08)	24.46 (22.73–26.19)	23.61 (21.77–25.45)	25.55 (23.68–27.41)

CI = confidence interval, KNHANES = Korea National Health and Nutrition Examination Survey, MASLD = metabolic dysfunction-associated steatotic liver disease.

### 3.2. Trends in MASLD prevalence by household income

**Figure 1.**
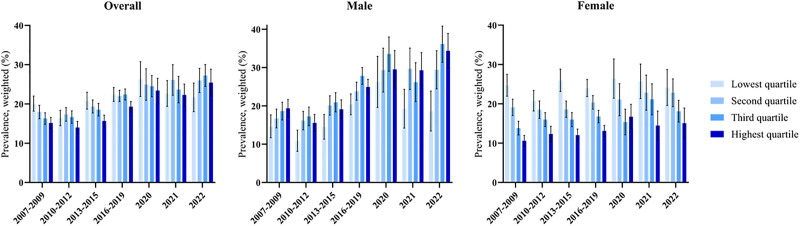
Trends in MASLD prevalence among Korean adults by household income levels (2007–2022). MASLD = metabolic dysfunction-associated steatotic liver disease.

The prevalence of MASLD increased overall from 20.08% (95% CI, 18.17–22.00) in 2007 to 2009 to 21.69% (95% CI, 18.04–25.33) in 2022. Across all household income levels, upward trends in prevalence were observed, with particularly marked increases in the medium-high and high-income groups, where prevalence increased from 16.30% to 27.25% and from 15.15% to 25.44%, respectively. In the low- and medium-low-income groups, prevalence also increased from 20.08% to 21.69% and from 17.95% to 25.98%, respectively. Overall, the lowest prevalence was consistently found in the high-income group (Fig. 1).

### 3.3. Sex- and age-specific differences in MASLD prevalence by household income

Among females, prevalence was highest in the low-income group (24.46% [95% CI, 23.07–25.86]) and lowest in the high-income group (13.67% [95% CI, 12.59–14.75]). Statistically significant β coefficients, reflecting upward temporal trends, were observed in the medium-high and high-income female groups prior to the pandemic. Among males, prevalence was highest in the low-income group (25.48% [95% CI, 24.05–26.92]) and lowest in the high-income group (17.98% [95% CI, 16.25–19.71]). Significant β coefficients were identified across all male income groups before the pandemic, with additional increases were observed in the medium-high and high-income groups during the pandemic. By contrast, in the low-income male group, the β_diff_ (−3.34 [95% CI, −5.70 to −0.99]) indicated a slower increase in prevalence compared with the pre-pandemic period (Tables 2 and S1, Supplemental Digital Content, https://links.lww.com/MD/Q376). Age-related differences were also notable, with the highest prevalence of SLD, defined by the HSI and SLD with metabolic comorbidities, occurring in middle-aged adults, particularly those aged 40 to 49 and 50 to 59 (Tables S2 and S3, Supplemental Digital Content, https://links.lww.com/MD/Q376).

**Table 2:** Trends in the prevalence of MASLD, stratified by household income group and β-coefficients of odds ratios before and during the COVID-19 pandemic (weighted % [95% CI]).

Variables	Household income level	Total	Before the pandemic				During the pandemic			Trends before the pandemic, β (95% CI)	Trends in the pandemic, β (95% CI)	β_diff_ between 2007–2019 and 2020–2022 (95% CI)
			2007–2009	2010–2012	2013–2015	2016–2019	2020	2021	2022			
Overall	Low	21.50 (20.37–22.63)	20.08 (18.17–22.00)	16.42 (14.47–18.37)	20.84 (18.70–22.97)	22.42 (20.66–24.17)	26.34 (21.92–30.77)	22.68 (19.41–25.95)	21.69 (18.04–25.33)	**1.15 (0.32–1.98**)	−0.57 (−1.90 to 0.76)	−**1.72** (−**3.29 to −0.15**)
	Low-medium	21.96 (20.93–22.99)	17.95 (16.23–19.67)	17.34 (15.61–19.08)	19.35 (17.69–21.01)	22.06 (20.64–23.48)	24.95 (20.95–28.95)	26.11 (22.22–30.01)	25.98 (22.93–29.04)	**1.46 (0.75–2.17**)	**1.30 (0.16–2.44**)	−0.16 (−1.50 to 1.17)
	Medium-high	21.64 (20.74–22.54)	16.30 (14.83–17.76)	16.66 (15.07–18.26)	18.54 (16.94–20.14)	22.42 (21.02–23.81)	24.52 (21.82–27.23)	23.66 (20.28–27.04)	27.25 (24.46–30.04)	**2.05 (1.41–2.70**)	**1.37 (0.33–2.41**)	−0.68 (−1.90 to 0.54)
	High	19.85 (18.90–20.80)	15.15 (13.71–16.58)	13.98 (12.39–15.56)	15.65 (14.16–17.15)	19.30 (17.97–20.63)	23.40 (20.27–26.52)	22.27 (19.47–25.07)	25.44 (22.01–28.86)	**1.47 (0.85–2.09**)	**1.72 (0.52–2.91**)	0.25 (−1.10 to 1.59)
Sex												
Male	Low	17.98 (16.25–19.71)	14.66 (11.66–17.67)	10.90 (8.12–13.68)	14.55 (11.32–17.78)	20.39 (17.66–23.12)	26.26 (19.58–32.93)	19.26 (14.22–24.30)	18.65 (13.42–23.87)	**2.11 (0.82–3.41**)	−1.23 (−3.20 to 0.74)	−**3.34** (−**5.70 to −0.99**)
	Low-medium	23.51 (22.00–25.02)	16.72 (14.27–19.17)	16.16 (13.75–18.58)	20.10 (17.57–22.63)	23.83 (21.47–26.20)	29.34 (23.60–35.07)	29.68 (24.27–35.10)	29.42 (24.45–34.40)	**2.57 (1.49–3.65**)	1.73 (−0.07 to 3.54)	−0.83 (−2.93 to 1.26)
	Medium-high	26.19 (24.75–27.64)	18.64 (16.33–20.96)	17.27 (14.84–19.70)	20.94 (18.44–23.44)	27.82 (25.62–30.02)	33.53 (29.05–38.00)	26.18 (21.10–31.26)	36.10 (31.36–40.83)	**3.19 (2.17–4.21**)	**1.78 (0.05–3.50**)	−1.41 (−3.42 to 0.59)
	High	25.48 (24.05–26.92)	19.35 (17.05–21.66)	15.54 (13.31–17.78)	19.11 (16.66–21.56)	24.93 (22.95–26.90)	29.52 (24.63–34.42)	29.28 (24.63–33.92)	34.36 (29.81–38.90)	**2.13 (1.16–3.09**)	**2.80 (1.15–4.45**)	0.67 (−1.24 to 2.58)
Female	Low	24.46 (23.07–25.86)	24.72 (21.94–27.51)	20.79 (18.18–23.40)	25.98 (23.13–28.83)	24.03 (21.86–26.20)	26.43 (21.45–31.41)	25.71 (21.32–30.10)	24.15 (19.58–28.72)	0.29 (−0.83 to 1.41)	−0.03 (−1.68 to 1.61)	−0.32 (−2.31 to 1.67)
	Low-medium	20.51 (19.35–21.66)	19.11 (17.03–21.19)	18.51 (16.28–20.74)	18.62 (16.54–20.70)	20.39 (18.67–22.11)	21.06 (16.97–25.15)	22.85 (18.37–27.32)	22.81 (19.28–26.35)	0.42 (−0.44 to 1.28)	0.90 (−0.42 to 2.22)	0.49 (−1.09 to 2.06)
	Medium-high	16.90 (15.88–17.92)	13.81 (12.08–15.55)	15.99 (14.23–17.75)	16.00 (14.20–17.79)	16.75 (15.16–18.35)	15.38 (12.11–18.65)	21.15 (17.21–25.09)	18.13 (15.37–20.90)	**0.87 (0.13–1.62**)	0.99 (−0.10 to 2.08)	0.12 (−1.20 to 1.44)
	High	13.67 (12.59–14.75)	10.58 (9.16–11.99)	12.32 (10.36–14.29)	12.02 (10.45–13.58)	13.09 (11.65–14.52)	16.72 (13.56–19.87)	14.45 (10.72–18.18)	15.06 (11.17–18.94)	**0.73 (0.07–1.38**)	0.34 (−1.01 to 1.69)	−0.39 (−1.88 to 1.11)
Age (yr)												
19–29	Low	18.97 (15.25–22.70)	12.71 (7.27–18.14)	11.18 (4.89–17.48)	12.77 (5.79–19.76)	16.05 (10.97–21.13)	31.20 (14.37–48.03)	22.51 (10.80–34.21)	24.53 (14.31–34.75)	1.13 (−1.27 to 3.53)	1.72 (−2.33 to 5.77)	0.59 (−4.11 to 5.30)
	Low-medium	18.22 (15.84–20.61)	11.00 (7.82–14.18)	12.81 (8.56–17.06)	16.95 (12.26–21.63)	17.83 (13.97–21.69)	28.14 (18.88–37.40)	20.72 (11.75–29.69)	23.83 (14.40–33.26)	**2.47 (0.85–4.09**)	1.16 (−2.09 to 4.42)	−1.30 (−4.94 to 2.33)
	Medium-high	17.78 (15.66–19.90)	13.89 (10.13–17.66)	12.29 (8.83–15.76)	15.79 (11.84–19.73)	16.94 (13.56–20.31)	24.53 (18.11–30.95)	18.73 (11.12–26.34)	21.44 (13.79–29.09)	1.28 (−0.32 to 2.88)	0.70 (−2.03 to 3.43)	−0.58 (−3.75 to 2.58)
	High	16.49 (14.43–18.55)	13.74 (9.85–17.63)	11.55 (8.07–15.03)	12.61 (9.44–15.78)	17.14 (13.84–20.44)	15.72 (9.72–21.73)	20.96 (15.06–26.86)	21.53 (13.20–29.87)	1.19 (−0.41 to 2.79)	1.87 (−0.88 to 4.63)	0.68 (−2.50 to 3.86)
30–39	Low	22.26 (17.47–27.06)	18.29 (11.19–25.39)	15.37 (8.48–22.25)	22.58 (13.23–31.93)	35.87 (26.29–45.44)	28.38 (9.69–47.07)	14.34 (0.00–33.97)	20.72 (7.31–34.13)	**5.67 (1.93–9.42**)	−5.70 (−11.53 to 0.13)	−**11.38** (−**18.30 to −4.45**)
	Low-medium	23.96 (21.60–26.32)	16.87 (13.55–20.19)	20.02 (16.34–23.70)	20.77 (16.71–24.84)	26.95 (23.35–30.56)	27.12 (17.28–36.96)	33.82 (21.62–46.02)	28.31 (19.83–36.80)	**3.11 (1.54–4.69**)	1.02 (−2.03 to 4.07)	−2.10 (−5.52 to 1.33)
	Medium-high	23.97 (22.05–25.88)	15.25 (12.71–17.80)	17.37 (14.21–20.52)	17.07 (14.20–19.94)	25.52 (22.42–28.62)	30.47 (23.65–37.28)	29.91 (22.69–37.12)	34.33 (27.82–40.84)	**3.00 (1.73–4.27**)	**2.60 (0.25–4.96**)	−0.40 (−3.07 to 2.27)
	High	20.17 (18.06–22.27)	14.73 (12.28–17.19)	12.05 (8.79–15.32)	16.20 (12.68–19.71)	21.15 (18.00–24.30)	23.07 (15.15–30.98)	21.59 (14.37–28.81)	29.73 (23.69–35.77)	**2.24 (0.96–3.52**)	**2.44 (0.07–4.81**)	0.20 (−2.49 to 2.90)
40–49	Low	27.88 (23.51–32.26)	23.33 (17.08–29.59)	16.47 (10.10–22.84)	18.99 (11.97–26.00)	27.36 (21.09–33.62)	46.52 (30.34–62.70)	36.53 (20.23–52.84)	30.96 (12.57–49.34)	1.41 (−1.43 to 4.25)	0.69 (−5.29 to 6.67)	−0.72 (−7.34 to 5.89)
	Low-medium	24.38 (22.04–26.73)	21.50 (17.39–25.60)	17.38 (13.94–20.82)	19.86 (16.10–23.63)	24.54 (21.12–27.96)	24.85 (16.00–33.70)	31.78 (22.38–41.17)	32.14 (25.14–39.14)	1.24 (−0.44 to 2.91)	**3.00 (0.36–5.64**)	1.76 (−1.36 to 4.89)
	Medium-high	23.28 (21.35–25.21)	16.99 (14.29–19.69)	19.07 (15.68–22.46)	20.31 (16.96–23.67)	24.37 (21.46–27.27)	24.95 (18.62–31.29)	26.43 (19.77–33.09)	29.18 (22.70–35.66)	**2.35 (1.07–3.63**)	1.61 (−0.73 to 3.95)	−0.74 (−3.40 to 1.92)
	High	20.54 (18.76–22.33)	14.21 (11.82–16.59)	17.17 (14.10–20.24)	14.10 (11.38–16.83)	19.14 (16.51–21.77)	27.48 (21.16–33.79)	27.08 (20.77–33.40)	24.49 (18.50–30.48)	**1.23 (0.09–2.37**)	1.60 (−0.53 to 3.74)	0.37 (−2.05 to 2.78)
50–59	Low	27.16 (23.71–30.60)	27.09 (21.75–32.44)	19.75 (14.71–24.79)	24.98 (19.09–30.87)	24.67 (19.41–29.93)	35.06 (21.55–48.56)	33.94 (19.89–47.98)	26.56 (15.52–37.60)	−0.19 (−2.57 to 2.19)	0.47 (−3.61 to 4.54)	0.66 (−4.06 to 5.37)
	Low-medium	24.66 (22.39–26.93)	22.21 (18.31–26.10)	19.26 (15.05–23.47)	21.04 (17.50–24.59)	21.64 (18.39–24.88)	27.90 (19.28–36.53)	30.52 (21.49–39.54)	32.02 (24.05–40.00)	0.01 (−1.61 to 1.63)	**3.43 (0.62–6.23**)	**3.42 (0.18–6.65**)
	Medium-high	21.12 (19.17–23.07)	18.51 (14.89–22.14)	17.30 (14.24–20.36)	19.21 (15.71–22.71)	24.57 (21.48–27.65)	23.91 (17.28–30.54)	17.16 (11.53–22.80)	24.86 (18.66–31.07)	**2.18 (0.70–3.67**)	−0.52 (−2.80 to 1.77)	−2.70 (−5.42 to 0.03)
	High	21.07 (19.25–22.90)	17.05 (14.07–20.02)	13.49 (10.87–16.12)	18.74 (15.70–21.78)	19.39 (17.02–21.76)	25.26 (20.01–30.51)	20.43 (15.32–25.55)	27.59 (21.15–34.02)	**1.37 (0.18–2.56**)	1.99 (−0.21 to 4.20)	0.62 (−1.88 to 3.12)
≥60	Low	19.74 (18.55–20.93)	20.17 (17.86–22.48)	16.90 (14.76–19.04)	21.61 (19.19–24.03)	21.47 (19.60–23.34)	19.56 (16.03–23.09)	19.46 (15.80–23.12)	19.12 (14.96–23.28)	0.88 (−0.06 to 1.82)	−0.72 (−2.17 to 0.73)	−1.60 (−3.33 to 0.13)
	Low-medium	19.57 (18.13–21.02)	19.10 (16.05–22.15)	16.58 (14.07–19.08)	18.25 (15.64–20.87)	19.84 (17.79–21.88)	20.79 (16.13–25.45)	20.33 (16.17–24.50)	20.29 (16.22–24.37)	0.51 (−0.62 to 1.64)	0.08 (−1.47 to 1.62)	−0.43 (−2.34 to 1.48)
	Medium-high	20.99 (19.15–22.83)	18.81 (14.48–23.14)	17.19 (14.09–20.30)	21.28 (18.01–24.55)	18.81 (16.39–21.22)	18.86 (13.29–24.42)	23.39 (18.20–28.58)	25.00 (19.72–30.28)	0.33 (−1.14 to 1.80)	**2.35 (0.40–4.30**)	2.02 (−0.42 to 4.46)
	High	20.85 (18.69–23.02)	18.92 (14.30–23.53)	15.77 (12.26–19.28)	17.18 (13.78–20.58)	19.98 (16.76–23.19)	24.39 (18.42–30.35)	22.22 (15.44–28.99)	21.86 (16.43–27.29)	0.64 (−1.07 to 2.35)	0.19 (−2.02 to 2.40)	−0.45 (−3.24 to 2.35)
Region of residence												
Urban	Low	21.53 (20.18–22.87)	20.53 (18.12–22.95)	16.52 (14.14–18.90)	21.04 (18.53–23.56)	22.69 (20.70–24.68)	25.58 (20.63–30.53)	23.00 (18.85–27.14)	20.86 (16.50–25.21)	**1.11 (0.11–2.10**)	−0.81 (−2.38 to 0.77)	−**1.91** (−**3.77 to −0.05**)
	Low-medium	21.79 (20.60–22.98)	18.27 (16.21–20.33)	16.73 (14.78–18.68)	18.96 (17.06–20.85)	21.70 (20.12–23.28)	25.37 (20.83–29.91)	26.09 (21.54–30.64)	25.14 (21.67–28.61)	**1.30 (0.48–2.12**)	1.12 (−0.17 to 2.42)	−0.18 (−1.71 to 1.35)
	Medium-high	21.23 (20.23–22.22)	16.57 (14.93–18.21)	15.85 (14.14–17.56)	17.94 (16.12–19.75)	21.75 (20.27–23.24)	24.62 (21.68–27.56)	22.94 (19.23–26.65)	26.39 (23.39–29.39)	**1.82 (1.12–2.53**)	**1.22 (0.10–2.34**)	−0.60 (−1.92 to 0.72)
	High	19.57 (18.54–20.60)	15.32 (13.77–16.88)	13.80 (12.06–15.55)	15.42 (13.85–16.99)	18.74 (17.35–20.12)	22.89 (19.55–26.22)	21.90 (18.88–24.92)	24.78 (21.12–28.45)	**1.25 (0.58–1.92**)	**1.70 (0.43–2.97**)	0.45 (−0.98 to 1.88)
Rural	Low	21.44 (19.35–23.52)	18.90 (16.12–21.69)	16.22 (12.86–19.58)	20.14 (16.31–23.97)	21.50 (17.86–25.13)	28.68 (19.25–38.11)	21.82 (16.30–27.35)	24.66 (18.67–30.65)	1.17 (−0.30 to 2.64)	0.25 (−2.13 to 2.64)	−0.92 (−3.72 to 1.88)
	Low-medium	22.73 (20.95–24.51)	16.68 (14.20–19.17)	19.65 (15.92–23.38)	21.11 (17.85–24.36)	23.91 (20.71–27.12)	22.82 (16.11–29.53)	26.21 (20.39–32.03)	29.55 (24.44–34.65)	**2.31 (1.00–3.62**)	**2.06 (0.04–4.08**)	−0.25 (−2.66 to 2.16)
	Medium-high	24.13 (21.88–26.39)	15.00 (11.93–18.07)	20.96 (16.77–25.15)	21.70 (18.33–25.06)	27.31 (23.40–31.22)	23.79 (17.33 to 30.25)	27.97 (20.59–35.35)	32.44 (23.88–41.01)	**3.75 (2.19–5.32**)	2.06 (−0.98 to 5.10)	−1.69 (−5.11 to 1.72)
	High	22.10 (19.72–24.48)	14.04 (10.49–17.59)	14.90 (11.10–18.70)	17.32 (12.72–21.93)	24.85 (20.26–29.43)	29.17 (20.47–37.87)	25.55 (19.34–31.76)	30.67 (23.96–37.39)	**3.39 (1.57–5.21**)	1.44 (−1.31 to 4.19)	−1.95 (−5.24 to 1.35)
Level of education												
Middle school or lower education	Low	22.13 (20.87–23.39)	22.94 (20.67–25.21)	18.26 (15.96–20.56)	22.82 (20.21–25.44)	22.87 (20.81–24.94)	23.17 (18.41–27.93)	22.35 (18.14–26.55)	22.93 (18.35–27.50)	0.40 (−0.59 to 1.38)	−0.06 (−1.66 to 1.54)	−0.45 (−2.33 to 1.42)
	Low-medium	21.39 (20.05–22.74)	22.08 (19.18–24.97)	18.79 (16.09–21.50)	21.75 (18.95–24.56)	22.31 (19.94–24.69)	21.74 (16.87–26.62)	21.53 (16.35–26.72)	21.90 (17.75–26.06)	0.29 (−0.92 to 1.49)	−0.15 (−1.73 to 1.43)	−0.44 (−2.42 to 1.54)
	Medium-high	23.46 (21.67–25.25)	20.49 (17.43–23.55)	19.61 (16.74–22.47)	23.98 (20.52–27.43)	25.34 (22.08–28.60)	20.49 (13.63–27.36)	24.83 (18.47–31.20)	35.42 (26.21–44.63)	**1.85 (0.43–3.26**)	**3.03 (0.13–5.94**)	1.19 (−2.04 to 4.42)
	High	21.30 (19.06–23.54)	17.81 (14.10–21.52)	16.99 (13.41–20.57)	21.21 (16.22–26.19)	26.43 (21.58–31.28)	26.30 (17.68–34.92)	17.60 (9.48–25.73)	29.38 (17.78–40.97)	**2.89 (0.97–4.81**)	−0.42 (−4.13 to 3.29)	−3.31 (−7.49 to 0.86)
College or higher education	Low	20.74 (18.90–22.59)	15.34 (12.03–18.66)	13.58 (10.13–17.03)	17.96 (14.39–21.52)	21.88 (19.11–24.65)	29.35 (22.63–36.07)	23.03 (17.77–28.30)	20.51 (15.60–25.42)	**2.49 (1.12–3.87**)	−1.13 (−3.07 to 0.81)	−**3.62** (−**6.00 to −1.25**)
	Low-medium	22.17 (20.88–23.45)	15.71 (13.73–17.69)	16.77 (14.66–18.88)	18.42 (16.46–20.38)	21.98 (20.22–23.73)	25.97 (21.15–30.79)	27.61 (22.78–32.44)	27.18 (23.32–31.04)	**2.09 (1.25–2.94**)	**1.72 (0.30–3.15**)	−0.37 (−2.02 to 1.29)
	Medium-high	21.36 (20.35–22.36)	15.22 (13.58–16.87)	15.96 (14.10–17.81)	17.59 (15.81–19.37)	21.95 (20.45–23.46)	25.03 (22.15–27.91)	23.53 (19.82–27.25)	26.51 (23.55–29.47)	**2.23 (1.52–2.94**)	**1.21 (0.10–2.33**)	−1.02 (−2.34 to 0.30)
	High	19.73 (18.73–20.73)	14.76 (13.30–16.23)	13.51 (11.81–15.21)	15.13 (13.60–16.67)	18.75 (17.40–20.10)	23.23 (20.00–26.46)	22.53 (19.58–25.48)	25.27 (21.78–28.76)	**1.43 (0.79–2.07**)	**1.86 (0.64–3.09**)	0.43 (−0.95 to 1.81)
Stress level												
High-stress level	Low	25.12 (22.76–27.47)	22.40 (18.89–25.92)	16.89 (13.18–20.60)	25.62 (21.04–30.20)	24.98 (21.56–28.40)	35.37 (24.90–45.84)	22.84 (16.11–29.57)	28.93 (19.73–38.12)	1.58 (0.00–3.16)	0.14 (−2.98 to 3.25)	−1.45 (−4.94 to 2.04)
	Low-medium	23.10 (21.23–24.98)	18.39 (15.38–21.40)	19.97 (16.68–23.26)	20.26 (16.68–23.84)	26.33 (23.36–29.30)	29.26 (21.55–36.98)	25.38 (18.69–32.07)	22.00 (16.37–27.62)	**2.44 (1.09–3.80**)	−1.61 (−3.73 to 0.50)	−**4.06** (−**6.56 to −1.55**)
	Medium-high	24.58 (22.81–26.36)	19.10 (16.12–22.08)	17.63 (14.49–20.77)	17.62 (14.57–20.66)	25.44 (22.79–28.08)	26.48 (21.18–31.78)	28.58 (21.64–35.52)	33.03 (27.63–38.43)	**1.99 (0.72–3.26**)	**2.53 (0.50–4.55**)	0.54 (−1.85 to 2.92)
	High	20.90 (19.04–22.77)	14.15 (11.51–16.78)	15.78 (12.59–18.96)	18.06 (14.81–21.31)	22.99 (20.33–25.64)	25.42 (18.71–32.13)	17.90 (13.15–22.64)	28.00 (21.86–34.13)	**2.95 (1.74–4.15**)	0.75 (−1.45 to 2.95)	−2.20 (−4.70 to 0.31)
Low-stress level	Low	20.23 (18.91–21.55)	19.08 (16.83–21.34)	16.22 (14.04–18.41)	18.98 (16.61–21.35)	21.48 (19.47–23.49)	23.01 (17.61–28.42)	22.64 (18.73–26.56)	19.67 (15.83–23.51)	**1.03 (0.08–1.99**)	−0.61 (−2.07 to 0.85)	−1.64 (−3.39 to 0.10)
	Low-medium	21.53 (20.37–22.68)	17.75 (15.77–19.72)	16.41 (14.57–18.26)	19.03 (17.17–20.89)	20.38 (18.77–21.99)	23.26 (18.90–27.63)	26.41 (21.92–30.91)	27.26 (23.77–30.75)	**1.10 (0.29–1.90**)	**2.37 (1.07–3.66**)	1.27 (−0.25 to 2.80)
	Medium-high	20.51 (19.45–21.57)	15.16 (13.49–16.82)	16.32 (14.56–18.09)	18.86 (17.02–20.70)	21.22 (19.59–22.85)	23.69 (20.20–27.19)	21.88 (18.19–25.57)	24.90 (21.40–28.40)	**2.08 (1.34–2.83**)	0.92 (−0.35 to 2.19)	−1.16 (−2.63 to 0.31)
	High	19.46 (18.38–20.54)	15.50 (13.79–17.22)	13.25 (11.53–14.97)	14.83 (13.08–16.58)	17.91 (16.47–19.34)	22.56 (19.09–26.04)	23.82 (20.25–27.39)	24.52 (20.86–28.17)	**0.92 (0.22–1.63**)	**2.09 (0.79–3.38**)	1.16 (−0.31 to 2.64)
Drinking status												
Nondrinker	Low	24.44 (22.74–26.13)	26.53 (23.24–29.82)	21.61 (18.47–24.74)	27.40 (23.99–30.81)	26.24 (23.51–28.98)	26.88 (20.54–33.22)	21.61 (17.20–26.02)	21.76 (16.62–26.89)	0.47 (−0.90 to 1.83)	−1.88 (−3.81 to 0.05)	−2.35 (−4.71 to 0.01)
	Low-medium	24.84 (22.93–26.74)	23.70 (20.19–27.21)	20.84 (17.16–24.53)	22.09 (18.93–25.26)	25.15 (22.33–27.97)	25.27 (19.17–31.36)	28.03 (21.56–34.49)	27.12 (22.31–31.93)	0.60 (−0.84 to 2.03)	0.88 (−1.10 to 2.85)	0.28 (−2.16 to 2.72)
	Medium-high	24.15 (22.27–26.04)	21.55 (18.35–24.75)	24.14 (20.23–28.05)	25.43 (21.74–29.12)	22.93 (20.16–25.70)	24.79 (18.66–30.93)	23.25 (17.54–28.96)	26.82 (20.39–33.26)	0.52 (−0.85 to 1.89)	1.00 (−1.28 to 3.28)	0.48 (−2.18 to 3.14)
	High	19.51 (17.66–21.36)	19.48 (16.17–22.80)	17.79 (14.17–21.41)	19.69 (16.17–23.22)	18.89 (16.12–21.66)	20.21 (14.77–25.65)	20.17 (14.16–26.19)	19.71 (14.49–24.92)	0.01 (−1.37 to 1.39)	0.22 (−1.77 to 2.22)	0.22 (−2.21 to 2.64)
Drinks more than once per month	Low	19.48 (18.01–20.94)	16.17 (13.90–18.44)	13.47 (11.18–15.75)	16.44 (13.73–19.14)	19.96 (17.72–22.20)	25.91 (20.44–31.39)	23.62 (18.63–28.61)	21.64 (16.63–26.65)	**1.47 (0.45–2.49**)	0.31 (−1.49 to 2.11)	−1.16 (−3.22 to 0.90)
	Low-medium	20.83 (19.68–21.98)	15.99 (14.22–17.76)	16.20 (14.35–18.06)	18.36 (16.34–20.39)	21.02 (19.38–22.66)	24.80 (20.33–29.28)	25.13 (20.53–29.74)	25.48 (21.43–29.53)	**1.75 (0.98–2.52**)	1.40 (−0.01 to 2.82)	−0.35 (−1.96 to 1.26)
	Medium-high	21.01 (19.98–22.03)	14.88 (13.26–16.51)	15.04 (13.29–16.78)	16.83 (15.15–18.51)	22.28 (20.68–23.89)	24.45 (21.45–27.45)	23.77 (19.89–27.64)	27.36 (24.05–30.67)	**2.45 (1.73–3.18**)	**1.46 (0.25–2.68**)	−0.99 (−2.40 to 0.42)
	High	19.92 (18.90–20.94)	14.28 (12.70–15.86)	13.16 (11.46–14.85)	14.87 (13.24–16.50)	19.37 (17.92–20.82)	24.02 (20.75–27.28)	22.83 (19.75–25.90)	26.59 (22.83–30.35)	**1.77 (1.09–2.46**)	**2.04 (0.74–3.34**)	0.27 (−1.20 to 1.74)
Smoking status												
Nonsmoker	Low	24.10 (22.57–25.63)	23.03 (20.43–25.64)	20.05 (17.38–22.73)	25.47 (22.70–28.24)	24.39 (22.18–26.59)	29.71 (23.58–35.85)	24.06 (19.79–28.32)	22.72 (17.98–27.47)	1.16 (−0.10 to 2.43)	−0.02 (−2.00 to 1.97)	−1.18 (−3.53 to 1.17)
	Low-medium	22.00 (20.63–23.37)	19.32 (17.17–21.46)	18.24 (15.99–20.49)	19.73 (17.52–21.94)	22.68 (20.87–24.49)	23.65 (18.53–28.77)	25.15 (19.49–30.81)	24.80 (20.88–28.72)	**1.69 (0.67–2.71**)	**1.99 (0.12–3.86**)	0.30 (−1.83 to 2.43)
	Medium-high	18.92 (17.82–20.02)	14.85 (12.97–16.73)	16.43 (14.48–18.38)	17.08 (15.18–18.97)	19.67 (18.03–21.31)	19.29 (15.67–22.91)	21.52 (17.51–25.52)	21.74 (18.56–24.92)	**2.78 (1.76–3.81**)	**1.95 (0.25–3.66**)	−0.83 (−2.82 to 1.16)
	High	16.33 (15.27–17.40)	13.05 (11.46–14.64)	13.95 (11.85–16.05)	13.32 (11.67–14.97)	15.71 (14.16–17.26)	18.78 (15.59–21.96)	18.18 (14.70–21.66)	19.17 (15.43–22.92)	**2.48 (1.44–3.53**)	**2.50 (0.75–4.26**)	0.02 (−2.02 to 2.06)
Smoker or ex-smoker	Low	17.97 (16.35–19.58)	16.56 (13.61–19.52)	11.92 (9.46–14.38)	14.94 (11.87–18.01)	19.49 (16.80–22.17)	22.27 (16.16–28.38)	20.60 (15.21–25.98)	20.00 (14.59–25.42)	0.92 (−0.16 to 2.00)	−1.01 (−2.75 to 0.72)	−1.93 (−3.98 to 0.11)
	Low-medium	21.91 (20.41–23.41)	16.48 (14.09–18.87)	16.32 (13.72–18.92)	18.87 (16.32–21.42)	21.25 (19.12–23.37)	26.62 (21.37–31.86)	27.30 (21.91–32.69)	27.59 (22.16–33.01)	**1.22 (0.32–2.11**)	0.78 (−0.70 to 2.26)	−0.44 (−2.16 to 1.29)
	Medium-high	24.94 (23.55–26.34)	17.79 (15.51–20.07)	16.91 (14.40–19.42)	20.37 (17.70–23.03)	25.87 (23.62–28.11)	31.64 (27.10–36.17)	26.48 (21.84–31.11)	33.99 (29.32–38.66)	**1.53 (0.73–2.32**)	0.85 (−0.35 to 2.06)	−0.67 (−2.11 to 0.77)
	High	24.46 (22.91–26.01)	17.70 (15.23–20.18)	14.01 (11.82–16.20)	18.97 (16.32–21.62)	24.15 (21.98–26.32)	29.52 (23.98–35.07)	28.08 (22.92–33.24)	32.98 (28.29–37.67)	**0.77 (0.06–1.49**)	0.97 (−0.34 to 2.28)	0.19 (−1.29 to 1.68)

β values were multiplied by 100 due to their small magnitudes.

The values in bold font represent significant variance (*P* < .05).

CI = confidence interval, KNHANES = Korea National Health and Nutrition Examination Survey, MASLD = metabolic dysfunction-associated steatotic liver disease.

### 3.4. Risk factors associated with MASLD

Factors associated with an increased risk of MASLD across income levels were identified. Males, middle-aged individuals, high-stress levels, and smoking status were statistically significant risk factors for MASLD. Participants with a college education or higher exhibited a greater likelihood of MASLD than those with a middle school or lower education. Males and current or former smokers tended to have a higher risk of MASLD compared to females and nonsmokers across all income levels. Compared with the high-income group, MASLD were significantly higher in the medium-high (aOR, 1.15 [95% CI, 1.08–1.23]), low-medium (aOR, 1.18 [95% CI, 1.10–1.26]), and low-income groups (aOR, 1.18 [95% CI, 1.10–1.27]) during the overall study period. Furthermore, the aORs for males, using females as the reference group, increased with the higher income levels. In addition, the aORs for males were statistically significant compared with females, with males in the high-income level showed a significantly higher risk of MASLD (aOR, 2.00 [95% CI, 1.85–2.17]) compared to those in the low-income level (aOR, 0.68 [95% CI, 0.62–0.76]). Across all household income groups, the relative risk of MASLD in males compared to females increased after COVID-19 pandemic (Table 3). The aOR for males tended to decrease with lower income levels, whereas the aOR for females tended to increase as income levels decreased (Table S4, Supplemental Digital Content, https://links.lww.com/MD/Q376).

**Table 3:** Adjusted odds ratios in the prevalence of MASLD, stratified by household income levels before and during COVID-19 pandemic (weighted % [95% CI]).

Household income level	Variables	Overall (2007–2022)		Before pandemic (2007–2019)		During pandemic (2020–2022)		Ratio of aORs (95% CI) during the pandemic compared to before the pandemic (reference)	
		aOR (95% CI)	*P*-value	aOR (95% CI)	*P*-value	aOR (95% CI)	*P*-value	Adjusted ratio of aOR (95% CI)	*P*-value
Sex									
Low	Female	1.00 (ref)		1.00 (ref)		1.00 (ref)		1.00 (ref)	
	Male	**0.68 (0.62–0.76**)	**.001**	**0.64 (0.57–0.72**)	**<.001**	0.85 (0.69–1.05)	.135	**1.33 (1.04–1.69**)	**.021**
Low-medium	Female	1.00 (ref)		1.00 (ref)		1.00 (ref)		1.00 (ref)	
	Male	**1.11 (1.02–1.20**)	**.013**	1.03 (0.94–1.13)	.474	**1.42 (1.20–1.68**)	**<.001**	**1.37 (1.13–1.66**)	**.001**
Medium-high	Female	1.00 (ref)		1.00 (ref)		1.00 (ref)		1.00 (ref)	
	Male	**1.63 (1.51–1.76**)	**<.001**	**1.52 (1.40–1.66**)	**<.001**	**2.05 (1.73–2.42**)	**<.001**	**1.34 (1.11–1.62**)	**.002**
High	Female	1.00 (ref)		1.00 (ref)		1.00 (ref)		1.00 (ref)	
	Male	**2.00 (1.85–2.17**)	**<.001**	**1.88 (1.71–2.06**)	**<.001**	**2.39 (2.00–2.86**)	**<.001**	**1.27 (1.04–1.56**)	**.019**
Age (yr)									
Low	19–29	1.00 (ref)		1.00 (ref)		1.00 (ref)		1.00 (ref)	
	30–39	**1.48 (1.11–1.98**)	**.008**	**1.83 (1.31–2.54**)	**<.001**	0.84 (0.42–1.66)	.611	**0.46 (0.22–0.98**)	**.043**
	40–49	**1.48 (1.14–1.93**)	**.004**	**1.57 (1.16–2.14**)	**.004**	1.61 (0.92–2.81)	.095	1.02 (0.54–1.93)	.944
	50–59	**1.63 (1.28–2.08**)	**<.001**	**1.91 (1.45–2.53**)	**<.001**	1.14 (0.69–1.90)	.611	0.60 (0.33–1.07)	.081
	≥60	1.10 (0.90–1.36)	0.356	**1.40 (1.09–1.80**)	**0.009**	**0.56 (0.38–0.82**)	**0.003**	**0.40 (0.25–0.63**)	**<0.001**
Low-medium	19–29	1.00 (ref)		1.00 (ref)		1.00 (ref)		1.00 (ref)	
	30–39	**1.50 (1.27–1.78**)	**<.001**	**1.59 (1.31–1.93**)	**<.001**	1.24 (0.86–1.79)	.250	0.78 (0.52–1.18)	.235
	40–49	**1.42 (1.20–1.68**)	**<.001**	**1.46 (1.21–1.76**)	**<.001**	1.22 (0.86–1.74)	.265	0.84 (0.56–1.25)	.390
	50–59	**1.44 (1.22–1.71**)	**<.001**	**1.46 (1.21–1.78**)	**<.001**	1.31 (0.93–1.84)	.124	0.89 (0.60–1.32)	.567
	≥60	1.11 (0.95–1.29)	.183	1.19 (0.99–1.41)	.059	0.76 (0.55–1.04)	.084	**0.64 (0.44–0.92**)	**.015**
Medium-high	19–29	1.00 (ref)		1.00 (ref)		1.00 (ref)		1.00 (ref)	
	30–39	**1.46 (1.25–1.69**)	**<.001**	**1.43 (1.20–1.70**)	**<.001**	**1.66 (1.24–2.24**)	**<.001**	1.17 (0.83–1.65)	.376
	40–49	**1.51 (1.31–1.75**)	**<.001**	**1.56 (1.31–1.84**)	**<.001**	**1.38 (1.02–1.87**)	**.038**	0.89 (0.63–1.25)	.497
	50–59	**1.41 (1.22–1.64**)	**<.001**	**1.55 (1.30–1.84**)	**<.001**	1.03 (0.76–1.39)	.845	**0.67 (0.47–0.94**)	**.021**
	≥60	**1.27 (1.10–1.48**)	**.002**	**1.34 (1.12–1.59**)	**.001**	1.01 (0.75–1.36)	.946	0.76 (0.54–1.06)	.108
High	19–29	1.00 (ref)		1.00 (ref)		1.00 (ref)		1.00 (ref)	
	30–39	**1.25 (1.07–1.46**)	**.006**	1.20 (1.00–1.43)	.050	**1.41 (1.03–1.92**)	**.032**	1.18 (0.82–1.68)	.377
	40–49	**1.27 (1.10–1.48**)	**0.001**	**1.22 (1.04–1.44**)	**0.017**	**1.52 (1.11–2.09**)	**0.010**	1.24 (0.87–1.78)	0.234
	50–59	**1.33 (1.16–1.54**)	**<.001**	**1.31 (1.12–1.55**)	**.001**	1.33 (0.99–1.78)	.056	1.01 (0.72–1.42)	.943
	≥60	**1.32 (1.13–1.55**)	**<.001**	**1.32 (1.10–1.58**)	**.003**	1.20 (0.88–1.64)	.252	0.91 (0.64–1.31)	.616
Region of residence									
Low	Urban	1.00 (ref)		1.00 (ref)		1.00 (ref)		1.00 (ref)	
	Rural	0.94 (0.84–1.05)	.260	0.92 (0.81–1.04)	.160	1.03 (0.80–1.32)	.840	1.12 (0.85–1.49)	.425
Low-medium	Urban	1.00 (ref)		1.00 (ref)		1.00 (ref)		1.00 (ref)	
	Rural	1.09 (0.98–1.20)	.114	1.10 (0.98–1.24)	.093	1.06 (0.87–1.30)	.541	0.96 (0.77–1.21)	.753
Medium-high	Urban	1.00 (ref)		1.00 (ref)		1.00 (ref)		1.00 (ref)	
	Rural	**1.18 (1.06–1.32**)	**.002**	**1.20 (1.06–1.35**)	**.004**	1.18 (0.94–1.49)	.164	0.98 (0.76–1.28)	.894
High	Urban	1.00 (ref)		1.00 (ref)		1.00 (ref)		1.00 (ref)	
	Rural	1.11 (0.97–1.27)	.123	1.10 (0.94–1.28)	.225	**1.27 (1.01–1.59**)	**.044**	1.15 (0.88–1.52)	.310
Level of education									
Low	Middle school or lower education	1.00 (ref)		1.00 (ref)		1.00 (ref)		1.00 (ref)	
	College or higher education	**0.90 (0.81–1.00**)	**.047**	**0.84 (0.74–0.94**)	**.003**	1.11 (0.90–1.38)	.341	**1.33 (1.04–1.70**)	**.023**
Low-medium	Middle school or lower education	1.00 (ref)		1.01 (ref)		1.00 (ref)		1.00 (ref)	
	College or higher education	1.06 (0.97–1.16)	.185	0.97 (0.89–1.07)	.593	**1.40 (1.14–1.71**)	**.001**	**1.43 (1.15–1.79**)	**.002**
Medium-high	Middle school or lower education	1.00 (ref)		1.00 (ref)		1.00 (ref)		1.00 (ref)	
	College or higher education	**1.10 (1.01–1.20**)	**.039**	1.01 (0.92–1.12)	.809	**1.35 (1.09–1.66**)	**.006**	**1.33 (1.05–1.68**)	**.016**
High	Middle school or lower education	1.00 (ref)		1.00 (ref)		1.00 (ref)		1.00 (ref)	
	College or higher education	**1.16 (1.04–1.31**)	**.010**	1.08 (0.95–1.23)	.223	1.23 (0.95–1.60)	.119	1.14 (0.85–1.53)	.380
Stress level									
Low	High-stress level	1.00 (ref)		1.00 (ref)		1.00 (ref)		1.00 (ref)	
	Low-stress level	**1.26 (1.13–1.41**)	**<.001**	**1.20 (1.07–1.36**)	**.003**	**1.56 (1.20–2.03**)	**.001**	1.29 (0.97–1.73)	.083
Low-medium	High-stress level	1.00 (ref)		1.00 (ref)		1.00 (ref)		1.00 (ref)	
	Low-stress level	**1.15 (1.05–1.26**)	**.004**	**1.20 (1.08–1.33**)	**<.001**	1.01 (0.82–1.24)	.939	0.84 (0.67–1.06)	.150
Medium-high	High-stress level	1.00 (ref)		1.00 (ref)		1.00 (ref)		1.00 (ref)	
	Low-stress level	**1.26 (1.15–1.38**)	**<.001**	**1.22 (1.10–1.35**)	**<.001**	**1.37 (1.13–1.65**)	**.001**	1.12 (0.90–1.39)	.298
High	High-stress level	1.00 (ref)		1.00 (ref)		1.00 (ref)		1.00 (ref)	
	Low-stress level	**1.17 (1.06–1.29**)	**.001**	**1.23 (1.11–1.37**)	**<.001**	1.02 (0.83–1.26)	.820	0.83 (0.66–1.05)	.120
Drinking status									
Low	Nondrinker	1.00 (ref)		1.00 (ref)		1.00 (ref)		1.00 (ref)	
	Drinks more than once per month	**0.78 (0.70–0.86**)	**<.001**	**0.70 (0.63–0.78**)	**<.001**	1.16 (0.93–1.44)	.181	**1.66 (1.30–2.12**)	**<.001**
Low-medium	Nondrinker	1.00 (ref)		1.00 (ref)		1.00 (ref)		1.00 (ref)	
	Drinks more than once per month	0.96 (0.88–1.04)	.314	0.94 (0.85–1.04)	.254	1.07 (0.89–1.29)	.477	1.13 (0.92–1.40)	.246
Medium-high	Nondrinker	1.00 (ref)		1.00 (ref)		1.00 (ref)		1.00 (ref)	
	Drinks more than once per month	1.06 (0.97–1.16)	.234	0.98 (0.89–1.09)	.750	**1.31 (1.07–1.62**)	**.011**	**1.34 (1.06–1.68**)	**.014**
High	Nondrinker	1.00 (ref)		1.00 (ref)		1.00 (ref)		1.00 (ref)	
	Drinks more than once per month	**1.17 (1.07–1.29**)	**.001**	1.10 (0.98–1.22)	.102	**1.41 (1.17–1.70**)	**<.001**	**1.28 (1.03–1.60**)	**.024**
Smoking status									
Low	Nonsmoker	1.00 (ref)		1.00 (ref)		1.00 (ref)		1.00 (ref)	
	Smoker or ex-smoker	**0.82 (0.74–0.91**)	**<.001**	**0.80 (0.71–0.90**)	**<.001**	0.90 (0.71–1.14)	.379	1.12 (0.86–1.46)	.396
Low-medium	Nonsmoker	1.00 (ref)		1.00 (ref)		1.00 (ref)		1.00 (ref)	
	Smoker or ex-smoker	**1.35 (1.24–1.47**)	**<.001**	**1.30 (1.18–1.43**)	**<.001**	**1.56 (1.28–1.90**)	**<.001**	1.20 (0.96–1.49)	.107
Medium-high	Nonsmoker	1.00 (ref)		1.00 (ref)		1.00 (ref)		1.00 (ref)	
	Smoker or ex-smoker	**1.87 (1.73–2.03**)	**<.001**	**1.77 (1.61–1.94**)	**<.001**	**2.26 (1.94–2.65**)	**<.001**	**1.28 (1.07–1.54**)	**.007**
High	Nonsmoker	1.00 (ref)		1.00 (ref)		1.00 (ref)		1.00 (ref)	
	Smoker or ex-smoker	**1.97 (1.81–2.14**)	**<.001**	**1.89 (1.71–2.08**)	**<.001**	**2.17 (1.83–2.57**)	**<.001**	1.15 (0.95–1.40)	.157

The values in bold font represent a significant variance (*P* < .05).

aOR = adjusted odds ratio, CI = confidence interval, KNHANES = Korea National Health and Nutrition Examination Survey, MASLD = metabolic dysfunction-associated steatotic liver disease.

## 4. Discussion

### 4.1. Key findings

This study analyzed the trends of MASLD stratified by household income levels using data from KNHANES from 2007 to 2022 (n = 70,276). The prevalence of MASLD has increased from 2007 to 2022. Particularly, in the medium-high and high household income levels, prevalence increased from 16.30% (95% CI, 14.83–17.76) in 2007 to 2009 to 27.25% (95% CI, 24.46–30.04) in 2022 and from 15.15% (95% CI, 13.71–16.58) in 2007 to 2009 to 25.44% (95% CI, 22.01–28.86) in 2022, respectively. The lowest prevalence of MASLD was generally observed in the high-income level. Additionally, the trends in MASLD prevalence exhibited opposite patterns between males and females across household income levels. Among females, prevalence increased in the lower income levels, whereas among males, prevalence increased in the higher income levels. Furthermore, males, middle-aged individuals with high-stress levels, and current or ex-smokers were identified as statistically significant risk factors for MASLD. Overall, males had a higher risk of MASLD than females across all income levels.

### 4.2. Plausible underlying mechanisms

This study observed an increasing trend in MASLD prevalence in South Korea from 2007 to 2022. This trend might be associated with the rising obesity rates in South Korea,^[2]^ as MASLD is strongly linked to obesity.^[21]^ The westernization of lifestyles, a greater preference for unhealthy foods and snacks, and less physical activity contribute to rising obesity rates, and these might be associated with the observed increase in MASLD prevalence.^[22]^ Additionally, the rise of MASLD prevalence might be associated with the aging population. The risk of MASLD tended to be higher in middle-aged and older age groups as the accumulation of senescent cells contributes to the steatotic liver, and hepatocyte aging is closely associated with the progression of MASLD.^[23]^ Therefore, given that South Korea is among the fastest-aging countries,^[24]^ the increasing prevalence of MASLD may be attributed to demographic shifts, particularly the rising proportion of middle-aged and older adults.

The prevalence of MASLD was lowest among individuals with the highest income levels. Individuals with lower levels of educational attainment and household income often experience restrictions in accessing health knowledge and medical services, which could increase their risk of developing MASLD. Such groups may also encounter barriers in obtaining the information and resources necessary for disease prevention and management, hindering the early detection and appropriate treatment of MASLD.^[25]^ Additionally, individuals in the low household income level may experience food insecurity, which could serve as a barrier to maintaining a healthy diet and may increase the risk of obesity and MASLD.^[26]^

The prevalence of MASLD exhibited a sex-specific trend. It was higher among low-income females. Among males, the prevalence tended to be higher in high-income groups, despite the lowest prevalence being observed in the high-income level. This might indicate that rising income among males may not be associated with corresponding changes toward healthier lifestyles and dietary patterns. High-income males tended to prefer animal-based dietary patterns, which were positively associated with obesity, abdominal obesity, and hypercholesterolemia.^[27]^ Furthermore, high-income males were also associated with a greater tendency for alcohol consumption, which could elevate the risk of MASLD.^[28,29]^ The sex-specific trend may reflect unique sociocultural and economic contexts in South Korea, highlighting potential differences in lifestyle and health behaviors between males and females across income levels. Females may be more likely to experience pressure regarding body weight compared to males.^[30]^ Furthermore, females may allocate a greater proportion of income toward obesity prevention and adopting healthier diets, which could contribute to the lower prevalence of MASLD observed among high-income females.^[27]^

In this study, males, middle-aged individuals, high-stress levels, and smoking status were statistically significant risk factors for MASLD. Current or ex-smokers had a higher risk of MASLD compared to nonsmokers. Smoking may be associated with the progression of liver fibrosis and visceral fat accumulation, potentially increasing the risk of MASLD.^[29]^ In addition, higher stress may contribute to an increased risk of metabolic syndrome, which could be associated with MASLD.^[31]^

### 4.3. Comparison with previous studies

Compared to previous studies, our findings support existing research indicating that the prevalence of MASLD in South Korea is increasing across various sociodemographic groups and household income levels.^[32]^ Similar to our findings, previous studies emphasized that aging, obesity, decreased physical activity, unhealthy dietary habits, and socioeconomic factors increase the risk of MASLD development.^[33]^ MASLD was associated with participants’ obesity; therefore, the increase in obesity rates in South Korea is likely associated with the rising prevalence of MASLD.^[34]^ Similar to prior studies conducted in Asia, age dependency was identified as a major risk factor for chronic liver diseases including MASLD.^[35]^ Studies presented a U-shaped trend in age groups, with MASLD prevalence appearing higher among middle-aged populations, consistent with our findings.^[35]^

Furthermore, our results support that males have a higher risk of MASLD compared to females, consistent with previous studies. Males tend to have a higher risk of MASLD than females due to biological differences, which make males more susceptible to MASLD.^[36]^ Sex-specific genetic variants and gut microbiota also influence MASLD risk, highlighting the potential for developing personalized treatments based on individual genetic characteristics, which may contribute to the higher risk of MASLD in males.^[37]^ Similar to our findings, previous studies emphasized that a high income level has the lowest prevalence of MASLD. Previous research found that the prevalence of MASLD and other progressive liver diseases was lowest in the highest-income group and highest in the lowest-income group.^[38]^ This phenomenon could be associated to limited access to resources promote a healthy lifestyle, such as nutritious diets, among socioeconomically disadvantaged groups.^[39]^

While some previous studies analyzed the prevalence of MASLD, those have not examined the differences in socioeconomic factors across income levels. To the best of our knowledge, this is the first study to analyze MASLD prevalence by household income levels in South Korea.

### 4.4. Clinical and policy implications

This study emphasized the necessity of enhancing MASLD screening and monitoring efforts within the population of South Korea. This study indicated the importance of identifying differences in the associations between sociodemographic factors and MASLD. Monitoring and screening MASLD prevalence could enhance understanding of MASLD trends in South Korea and could help identify factors contributing to its progression. It is crucial to raise awareness among high-income males about the need for personalized lifestyle modifications and a healthy diet. Additionally, the high prevalence of MASLD among low-income females might be associated with food insecurity and limited access to a healthy diet.^[39]^ The government should enhance public awareness through nationwide campaigns and educational programs while expanding support for providing healthy meals to low-income individuals to promote healthy diets and lifestyles. Monitoring MASLD in vulnerable factors and populations is crucial for addressing public health. It is essential to identify MASLD prevalence among vulnerable groups and integrate these findings into the National Health Insurance strategic planning.

### 4.5. Strength and limitations

This study has several strengths. First, it used nationally representative data collected over 16 years to monitor trends in the MASLD prevalence stratified by household income level. Second, to the best of our knowledge, this is the first study to analyze MASLD prevalence by sociodemographic factors stratified by income levels. Third, we investigated the association between sociodemographic factors, household income levels, and MASLD, suggesting MASLD prevalence across income levels.

However, this study had several limitations. First, as a cross-sectional study, it could not track changes within the same individuals, limiting our ability to understand how factors such as aging, lifestyle changes, or urbanization affect the onset or progression of MASLD over time. The study utilized large-scale data to compensate for this limitation and suggested various compensatory mechanisms by referencing previous studies. However, to clarify causal relationships, future longitudinal studies are necessary. Second, MASLD was diagnosed using HSI instead of ultrasound or biopsy, the standard method for diagnosing MASLD. However, HSI is known to have high sensitivity and specificity and is considered the most suitable indicator for Asian populations.^[40]^ Third, the health and nutrition surveys were collected through self-reporting, which may lead to recall bias or inaccuracies. To minimize this, the study used the KNHANES, designed to reduce reporting errors, and analyzed data from a large-scale sample over an extended period. However, future research should consider supplementing self-reported data with medical records or genetic data to enhance the accuracy of health-related information. Fourth, since this study only used data from South Korea, there may be limitations in applying our findings to other ethnicities or countries. Similar follow-up studies using data from diverse countries are needed. Due to data limitations, certain factors could not be fully accounted for. However, the data used in this study is nationally representative, providing valuable insights into household income-related disparities in MASLD prevalence. Despite these findings, future research should incorporate additional nongenetic factors to provide a more comprehensive understanding.

## 5. Conclusion

This study is the first to analyze trends in MASLD stratified by household income levels using KNHANES data from 2007 to 2022, encompassing a total of 70,276 adults in South Korea. In this study, the prevalence of MASLD increased from 2007 to 2022, particularly in the medium-high and high household income levels. The prevalence trends of MASLD differed between males and females across household income levels. Among females, MASLD prevalence increased in the lower income levels, whereas among males, it increased in the higher income levels. Furthermore, males, middle-aged individuals, high-stress levels, and smoking status were statistically significant risk factors for MASLD. Additionally, the lowest MASLD prevalence was observed in the high household income level. These findings should be incorporated into public health planning, as they underscore the necessity for nationwide campaigns and programs to promote healthy dietary practices and enhance public awareness.

## Author contributions

**Conceptualization:** Yerin Cho, Hyunjee Kim, Jiyoung Hwang, Dong Keon Yon.

**Data curation:** Yerin Cho, Hyunjee Kim, Jiyoung Hwang, Dong Keon Yon.

**Formal analysis:** Yerin Cho, Hyunjee Kim, Jiyoung Hwang, Dong Keon Yon.

**Funding acquisition:** Jiyoung Hwang, Dong Keon Yon.

**Investigation:** Yerin Cho, Hyunjee Kim, Jiyoung Hwang, Dong Keon Yon.

**Methodology:** Yerin Cho, Hyunjee Kim, Jiyoung Hwang, Dong Keon Yon.

**Project administration:** Yerin Cho, Hyunjee Kim, Jiyoung Hwang, Dong Keon Yon.

**Resources:** Yerin Cho, Hyunjee Kim, Jiyoung Hwang, Dong Keon Yon.

**Software:** Yerin Cho, Hyunjee Kim, Jiyoung Hwang, Dong Keon Yon.

**Supervision:** Dong Keon Yon.

**Validation:** Hyunjee Kim, Dong Keon Yon.

**Visualization:** Yerin Cho, Hyunjee Kim, Jiyoung Hwang, Dong Keon Yon.

**Writing – original draft:** Yerin Cho, Hyunjee Kim, Jiyoung Hwang, Dong Keon Yon.

**Writing – review & editing:** Yerin Cho, Hyunjee Kim, Jinyoung Jeong, Jiyeon Oh, Jaeyu Park, Jaewon Kim, Jiyoung Hwang, Dong Keon Yon.

## Supplementary Material


