# Supplementary material for: Trends in metabolic dysfunction-associated steatotic liver disease by household income, 2007–2022: A national representative study in South Korea
Source: Medicine (Baltimore). 2025 Oct 24;104(43):e45296. doi: 10.1097/MD.0000000000045296 (PMC12558188; doi:10.1097/MD.0000000000045296)
Supplement: Supplementary file 1 [file medi-104-e45296-s001.pdf]

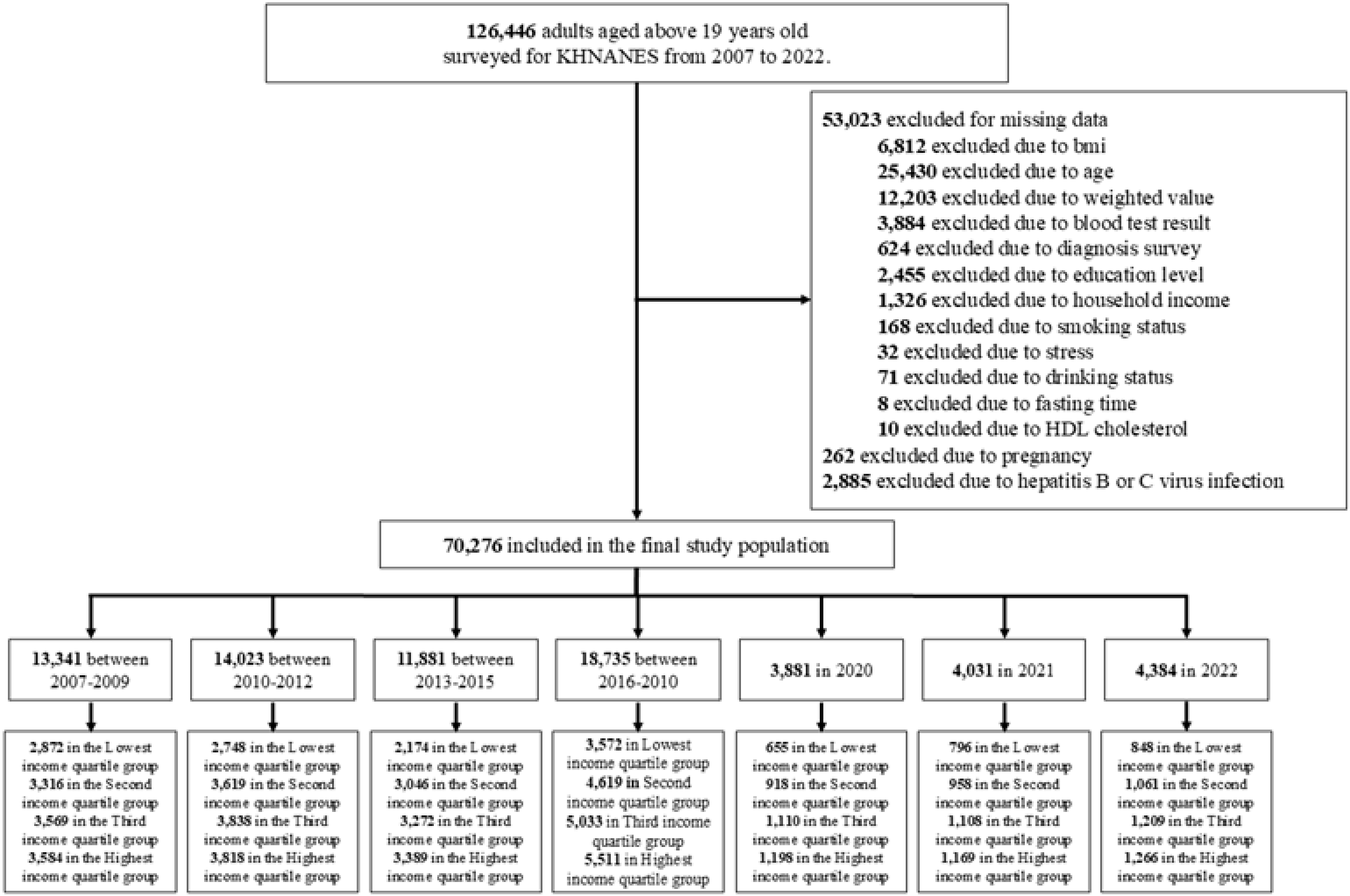
126,446 adults aged above 19 years old surveyed for KHNANES from 2007 to 2022.
53,023 excluded for missing data
6,812 excluded due to bmi
25,430 excluded due to age
12,203 excluded due to weighted value
3,884 excluded due to blood test result
624 excluded due to diagnosis survey
2,455 excluded due to education level
1,326 excluded due to household income
168 excluded due to smoking status
32 excluded due to stress
71 excluded due to drinking status
8 excluded due to fasting time
10 excluded due to HDL cholesterol
262 excluded due to pregnancy
2,885 excluded due to hepatitis B or C virus infection
70,276 included in the final study population
13,341 between 2007-2009
14,023 between 2010-2012
11,881 between 2013-2015
18,735 between 2016-2010
3,881 in 2020
4,031 in 2021
4,384 in 2022
2,872 in the Lowest income quartile group 3,316 in the Second income quartile group 3,569 in the Third income quartile group 3,584 in the Highest income quartile group
2,748 in the Lowest income quartile group 3,619 in the Second income quartile group 3,838 in the Third income quartile group 3,818 in the Highest income quartile group
2,174 in the Lowest income quartile group 3,046 in the Second income quartile group 3,272 in the Third income quartile group 3,389 in the Highest income quartile group
3,572 in Lowest income quartile group 4,619 in Second income quartile group 5,033 in Third income quartile group 5,511 in Highest income quartile group
655 in the Lowest income quartile group 918 in the Second income quartile group 1,110 in the Third income quartile group 1,198 in the Highest income quartile group
796 in the Lowest income quartile group 958 in the Second income quartile group 1,108 in the Third income quartile group 1,169 in the Highest income quartile group
848 in the Lowest income quartile group 1,061 in the Second income quartile group 1,209 in the Third income quartile group 1,266 in the Highest income quartile group
