# Supplementary material for: Trends in metabolic dysfunction-associated steatotic liver disease by household income, 2007–2022: A national representative study in South Korea
Source: Medicine (Baltimore). 2025 Oct 24;104(43):e45296. doi: 10.1097/MD.0000000000045296 (PMC12558188; doi:10.1097/MD.0000000000045296)
Supplement: Supplementary file 2 [file medi-104-e45296-s002.pdf]

Table S1. Baseline Characteristic of MASLD in Korean adults in KNHANES, based on the data from the KNHANES, 2007–2022

Overall, n	Total	2007-2009	2010-2012	2013-2015	2016-2019	2020	2021	2022
**Crude rate, n (%)**								
Total	13,492 (19.20)	2,303 (17.26)	2,201 (15.70)	2,175 (18.31)	3,922 (20.93)	932 (24.01)	900 (22.33)	1,059 (24.16)
Sex, n (%)								
Male	5,753 (19.72)	863 (16.15)	771 (15.62)	864 (17.60)	1,831 (23.12)	476 (28.33)	412 (23.82)	536 (28.00)
Female	7,739 (18.83)	1,440 (18.00)	1430 (16.51)	1,311 (18.80)	2,091 (19.33)	456 (20.72)	488 (21.21)	523 (21.17)
Age (years), n (%)								
20-29	1,177 (15.02)	187 (12.25)	155 (10.82)	181 (13.73)	339 (16.45)	119 (22.93)	86 (18.66)	110 (21.24)
30–39	2,148 (18.21)	385 (14.15)	354 (13.85)	314 (15.91)	676 (22.95)	138 (25.75)	119 (25.27)	162 (27.32)
40–49	2,534 (19.58)	457 (17.36)	404 (16.21)	378 (16.99)	749 (21.39)	177 (25.92)	174 (25.66)	195 (26.68)
50–59	2,814 (21.26)	489 (21.33)	481 (17.38)	504 (21.06)	765 (21.69)	189 (26.11)	171 (23.14)	215 (27.01)
≥60	4,819 (19.70)	785 (18.84)	807 (16.90)	798 (20.09)	1,393 (20.79)	309 (21.78)	350 (20.81)	377 (21.59)
Region of residence, n (%)								
Urban	10,410 (18.86)	1,684 (17.42)	1,702 (15.47)	1,726 (17.93)	3,082 (20.26)	736 (23.42)	679 (21.78)	801 (23.39)
Rural	3,082 (20.43)	619 (16.84)	499 (16.52)	449 (19.92)	840 (23.84)	196 (26.56)	221 (24.21)	258 (26.88)
Level of education, n (%)								
Middle school or lower education	4,947 (21.95)	1,130 (21.20)	959 (19.00)	849 (22.72)	1,262 (23.71)	234 (24.58)	251 (22.82)	262 (24.86)
College or higher education	8,545 (17.90)	1,173 (14.64)	1,242 (13.84)	1,326 (16.28)	2,660 (19.83)	698 (23.83)	649 (22.14)	797 (23.93)
Household income level, n (%)								
Low	2,872 (21.02)	591 (20.58)	481 (17.50)	466 (21.44)	811 (22.70)	162 (24.73)	175 (21.98)	186 (21.93)
Low-medium	3,562 (20.31)	618 (18.64)	617 (17.05)	591 (19.40)	1,024 (22.17)	229 (24.95)	224 (23.38)	259 (24.41)
Medium-high	3,672 (19.19)	562 (15.75)	615 (16.02)	590 (18.03)	1,073 (21.32)	268 (24.14)	257 (23.19)	307 (25.39)
High	3,386 (16.99)	532 (14.84)	488 (12.78)	528 (15.58)	1,014 (18.40)	273 (22.79)	244 (20.87)	307 (24.25)

Level of stress, n (%)								
High stress level	3,828 (21.00)	693 (18.56)	609 (16.92)	558 (19.58)	1,169 (23.74)	283 (26.80)	232 (23.13)	284 (26.72)
Low stress level	9,664 (18.57)	1,610 (16.76)	1,592 (15.27)	1,617 (17.91)	2,753 (19.93)	649 (22.97)	668 (22.06)	775 (23.34)
Drinking status, n (%)								
Non-drinker	4,594 (22.59)	894 (22.41)	815 (20.14)	786 (23.21)	1,219 (23.63)	275 (23.56)	297 (22.10)	308 (24.72)
Drinks more than once per month	8,898 (17.82)	1,409 (15.07)	1,386 (13.89)	1,389 (16.35)	2,703 (19.91)	657 (24.21)	603 (22.44)	751 (23.93)
Smoking status, n (%)								
Smoker or ex-smoker	5,213 (19.10)	830 (15.73)	754 (13.72)	785 (17.40)	1,596 (22.10)	410 (27.17)	365 (23.69)	473 (27.15)
Non-smoker	8,279 (19.26)	1,473 (18.27)	1,447 (16.97)	1,390 (18.86)	2,326 (20.20)	522 (22.01)	535 (21.49)	586 (22.18)
**Weighted rate (95% CI)**								
Total	21.12 (20.58 to 21.66)	2.22 (2.09 to 2.36)	2.14 (2.00 to 2.28)	2.32 (2.19 to 2.46)	3.16 (2.96 to 3.36)	3.65 (3.09 to 4.22)	3.60 (3.07 to 4.13)	4.02 (3.68 to 4.37)
Sex, weighted % (95% CI)								
Male	24.26 (23.46 to 25.06)	2.34 (2.15 to 2.53)	2.08 (1.89 to 2.27)	2.47 (2.27 to 2.68)	3.69 (3.42 to 3.96)	4.52 (3.77 to 5.28)	4.14 (3.44 to 4.83)	5.02 (4.51 to 5.53)
Female	17.97 (17.35 to 18.59)	2.11 (1.95 to 2.26)	2.19 (2.03 to 2.36)	2.18 (2.03 to 2.32)	2.63 (2.44 to 2.82)	2.79 (2.30 to 3.27)	3.07 (2.55 to 3.58)	3.02 (2.69 to 3.36)
Age (years), weighted % (95% CI)								
20-29	17.57 (16.33 to 18.80)	1.87 (1.56 to 2.17)	1.68 (1.37 to 1.98)	1.94 (1.61 to 2.26)	2.47 (2.14 to 2.80)	3.33 (2.47 to 4.19)	2.99 (2.23 to 3.75)	3.30 (2.55 to 4.05)
30–39	22.53 (21.32 to 23.73)	2.50 (2.19 to 2.80)	2.48 (2.14 to 2.82)	2.38 (2.07 to 2.70)	3.51 (3.14 to 3.89)	3.71 (2.81 to 4.62)	3.66 (2.78 to 4.55)	4.28 (3.47 to 5.08)
40–49	22.83 (21.64 to 24.01)	2.51 (2.23 to 2.79)	2.54 (2.23 to 2.86)	2.39 (2.12 to 2.66)	3.31 (2.97 to 3.65)	3.97 (3.09 to 4.84)	4.06 (3.08 to 5.05)	4.04 (3.33 to 4.75)
50–59	22.42 (21.28 to 23.55)	2.27 (2.00 to 2.54)	2.14 (1.88 to 2.40)	2.67 (2.38 to 2.96)	3.37 (3.03 to 3.71)	4.04 (3.12 to 4.96)	3.44 (2.75 to 4.14)	4.48 (3.78 to 5.18)
≥60	20.13 (19.31 to 20.94)	1.99 (1.78 to 2.20)	1.85 (1.67 to 2.03)	2.22 (2.02 to 2.43)	3.08 (2.82 to 3.35)	3.26 (2.65 to 3.87)	3.74 (3.07 to 4.42)	3.98 (3.47 to 4.49)

Region of residence, weighted % (95% CI)								
Urban	20.83 (20.22 to 21.44)	12.70 (12.00 to 13.40)	12.69 (11.98 to 13.40)	12.74 (12.07 to 13.41)	15.21 (14.24 to 16.19)	15.45 (13.09 to 17.82)	15.37 (13.11 to 17.63)	15.84 (14.61 to 17.07)
Rural	22.66 (21.52 to 23.81)	2.48 (2.03 to 2.93)	2.99 (2.38 to 3.61)	2.66 (2.14 to 3.18)	3.10 (2.50 to 3.70)	3.17 (1.98 to 4.36)	3.74 (2.47 to 5.01)	4.52 (3.04 to 6.00)
Level of education, weighted % (95% CI)								
Middle school or lower education	22.07 (21.27 to 22.87)	3.83 (3.48 to 4.18)	3.15 (2.86 to 3.44)	3.09 (2.82 to 3.37)	3.38 (3.08 to 3.69)	2.75 (2.18 to 3.31)	2.80 (2.24 to 3.36)	3.07 (2.59 to 3.54)
College or higher education	20.87 (20.24 to 21.51)	1.80 (1.68 to 1.93)	1.87 (1.72 to 2.03)	2.12 (1.97 to 2.28)	3.10 (2.88 to 3.32)	3.89 (3.26 to 4.53)	3.81 (3.21 to 4.42)	4.27 (3.85 to 4.70)
Household income level, weighted % (95% CI)								
Low	21.50 (20.37 to 22.63)	2.85 (2.51 to 3.19)	2.32 (1.99 to 2.65)	2.55 (2.22 to 2.87)	3.40 (3.03 to 3.78)	3.60 (2.65 to 4.56)	3.26 (2.53 to 3.98)	3.52 (2.87 to 4.18)
Low-medium	21.96 (20.93 to 22.99)	2.47 (2.19 to 2.75)	2.66 (2.35 to 2.97)	2.60 (2.33 to 2.88)	3.25 (2.95 to 3.55)	3.43 (2.65 to 4.21)	3.75 (2.93 to 4.56)	3.81 (3.23 to 4.38)
Medium-high	21.64 (20.74 to 22.54)	2.11 (1.88 to 2.33)	2.21 (1.97 to 2.46)	2.38 (2.13 to 2.64)	3.21 (2.92 to 3.51)	3.68 (2.96 to 4.39)	3.72 (2.99 to 4.44)	4.33 (3.74 to 4.92)
High	19.85 (18.90 to 20.80)	1.87 (1.64 to 2.11)	1.60 (1.39 to 1.82)	1.96 (1.74 to 2.19)	2.93 (2.63 to 3.22)	3.82 (2.99 to 4.66)	3.54 (2.80 to 4.28)	4.12 (3.51 to 4.73)
Level of stress, weighted % (95% CI)								
High stress level	23.13 (22.13 to 24.13)	2.52 (2.27 to 2.77)	2.34 (2.08 to 2.60)	2.39 (2.13 to 2.65)	3.74 (3.41 to 4.07)	4.39 (3.52 to 5.27)	3.45 (2.77 to 4.14)	4.29 (3.70 to 4.88)
Low stress level	20.37 (19.75 to 20.99)	2.11 (1.97 to 2.26)	2.06 (1.91 to 2.21)	2.30 (2.15 to 2.45)	2.94 (2.74 to 3.14)	3.38 (2.79 to 3.96)	3.66 (3.10 to 4.22)	3.92 (3.55 to 4.29)
Drinking status,weighted % (95% CI)								
Non-drinker	23.35 (22.41 to 24.30)	2.93 (2.66 to 3.20)	2.63 (2.36 to 2.89)	2.91 (2.64 to 3.19)	3.24 (2.96 to 3.53)	3.80 (3.03 to 4.57)	4.04 (3.29 to 4.78)	3.81 (3.31 to 4.30)
Drinks more than once per month	20.41 (19.81 to 21.02)	2.00 (1.86 to 2.14)	1.98 (1.83 to 2.13)	2.14 (1.99 to 2.28)	3.13 (2.91 to 3.35)	3.61 (3.02 to 4.20)	3.46 (2.91 to 4.01)	4.09 (3.68 to 4.50)

Smoking status, weighted % (95% CI)								
Smoker or ex-smoker	23.10 (22.30 to 23.89)	2.42 (2.21 to 2.63)	2.13 (1.94 to 2.33)	2.37 (2.17 to 2.56)	3.36 (3.10 to 3.62)	4.18 (3.47 to 4.90)	3.89 (3.26 to 4.53)	4.74 (4.20 to 5.28)
Non-smoker	19.56 (18.89 to 20.24)	2.07 (1.92 to 2.21)	2.14 (1.97 to 2.32)	2.29 (2.13 to 2.45)	2.99 (2.78 to 3.21)	3.24 (2.67 to 3.81)	3.37 (2.81 to 3.94)	3.46 (3.07 to 3.84)

Abbreviations: CI, confidence interval; MASLD, metabolic dysfunction–associated steatotic liver disease; KNHANES, Korea National Health and Nutrition Examination Survey.

Table S2. Crude rate of SLD evaluated using the HSI method in Korean adults based on data from the KNHANES, 2007–2022

Overall, n	Total	2007-2009	2010-2012	2013-2015	2016-2019	2020	2021	2022
**Crude rate, n (%)**								
Total	16,025 (22.80)	2,953 (22.13)	2,895 (20.64)	2,705 (22.77)	4,379 (23.37)	1,010 (26.02)	985 (24.44)	1,098 (25.05)
Sex, n (%)								
Male	7,389 (25.33)	1,274 (23.84)	1,209 (21.30)	1,233 (25.12)	2,125 (26.84)	527 (31.37)	459 (26.53)	562 (29.36)
Female	8,636 (21.01)	1,679 (20.99)	1,686 (20.20)	1,472 (21.11)	2,254 (20.84)	483 (21.94)	526 (22.86)	536 (21.70)
Age (years), n (%)								
20-29	1,431 (18.26)	257 (16.83)	215 (15.01)	231 (17.53)	385 (18.68)	131 (25.24)	096 (20.82)	116 (22.39)
30–39	2,735 (23.19)	560 (20.58)	534 (20.89)	423 (21.44)	763 (25.90)	151 (28.17)	133 (28.24)	171 (28.84)
40–49	3,162 (24.43)	645 (24.50)	570 (22.87)	518 (23.28)	851 (24.31)	190 (27.82)	186 (27.43)	202 (27.63)
50–59	3,369 (25.45)	616 (26.86)	647 (23.38)	615 (25.70)	873 (24.75)	207 (28.59)	186 (25.17)	225 (28.27)
≥60	5,328 (21.78)	875 (21.00)	929 (19.45)	918 (23.11)	1,507 (22.49)	331 (23.33)	384 (22.83)	384 (21.99)
Region of residence, n (%)								
Urban	12,412 (22.49)	2,184 (22.60)	2,258 (20.52)	2,135 (22.18)	3,446 (22.65)	804 (25.58)	751 (24.09)	834 (24.36)
Rural	3,613 (23.95)	769 (20.92)	637 (21.09)	570 (25.29)	933 (26.48)	206 (27.91)	234 (25.63)	264 (27.50)
Level of education, n (%)								
Middle school or lower education	5,528 (24.52)	1,294 (24.28)	1,131 (22.41)	969 (25.94)	1,347 (25.31)	246 (25.84)	277 (25.18)	264 (25.05)
College or higher education	10,497 (21.99)	1,659 (20.71)	1,764 (19.65)	1,736 (21.31)	3,032 (22.61)	764 (26.08)	708 (24.16)	834 (25.05)
Household income level, n (%)								
Low	3,154 (23.08)	661 (23.02)	549 (19.98)	536 (24.66)	861 (24.10)	173 (26.41)	186 (23.37)	188 (22.17)
Low-medium	4,196 (23.93)	786 (23.70)	793 (21.91)	728 (23.90)	1,134 (24.55)	241 (26.25)	240 (25.05)	274 (25.82)
Medium-high	4,436 (23.18)	755 (21.15)	826 (21.52)	748 (22.86)	1,212 (24.08)	293 (26.40)	288 (25.99)	314 (25.97)
High	4,239 (21.26)	751 (20.95)	727 (19.04)	693 (20.45)	1,172 (21.27)	303 (25.29)	271 (23.18)	322 (25.43)
Level of stress, n (%)								
High stress level	4,560 (25.02)	898 (24.06)	803 (22.31)	705 (24.74)	1,302 (26.44)	307 (29.07)	251 (25.02)	294 (27.66)

Low stress level	11,465 (22.03)	2,055 (21.39)	2,092 (20.07)	2,000 (22.15)	3,077 (22.28)	703 (24.89)	734 (24.24)	804 (24.21)
Drinking status, n (%)								
Non-drinker	4,884 (24.01)	955 (23.93)	878 (21.70)	842 (24.86)	1,297 (25.15)	292 (25.02)	311 (23.14)	309 (24.80)
Drinks more than once per month	11,141 (22.31)	1,998 (21.37)	2,017 (20.22)	1,863 (21.93)	3,082 (22.70)	718 (26.46)	674 (25.08)	789 (25.14)
Smoking status, n (%)								
Smoker or ex-smoker	6,712 (24.59)	1,210 (22.93)	1,172 (21.33)	1,117 (24.76)	1,857 (25.71)	454 (30.09)	404 (26.22)	498 (28.59)
Non-smoker	9,313 (21.67)	1,743 (21.62)	1,723 (20.20)	1,588 (21.55)	2,522 (21.91)	556 (23.44)	581 (23.33)	600 (22.71)

Abbreviations: CI, confidence interval; HSI, Hepatic Steatosis Index; KNHANES, Korea National Health and Nutrition Examination Survey; SLD, steatotic liver disease.

Table S3. Crude rate of Korean adults with both SLD and metabolic diseases based on data from the KNHANES, 2007–2022

Overall, n	Total	2007-2009	2010-2012	2013-2015	2016-2019	2020	2021	2022
**Crude rate, n (%)**								
Total	15,909 (22.64)	2,909 (21.81)	2,877 (20.52)	2,692 (22.66)	4,356 (23.25)	1,006 (25.92)	981 (24.34)	1,088 (24.82)
Sex, n (%)								
Male	7,341 (25.17)	1,257 (23.53)	1,200 (21.14)	1,229 (25.04)	2,113 (26.69)	525 (31.25)	459 (26.53)	558 (29.15)
Female	8,568 (20.84)	1,652 (20.66)	1,677 (20.09)	1,463 (20.98)	2,243 (20.74)	481 (21.85)	522 (22.69)	530 (21.46)
Age (years), n (%)								
20-29	1,397 (17.83)	251 (16.44)	212 (14.80)	225 (17.07)	374 (18.15)	128 (24.66)	094 (20.39)	113 (21.81)
30–39	2,691 (22.81)	539 (19.81)	528 (20.66)	421 (21.34)	756 (25.66)	150 (27.99)	131 (27.81)	166 (27.99)
40–49	3,136 (24.23)	634 (24.08)	564 (22.63)	514 (23.10)	847 (24.19)	190 (27.82)	186 (27.43)	201 (27.50)
50–59	3,359 (25.37)	610 (26.60)	645 (23.31)	614 (25.66)	873 (24.75)	207 (28.59)	186 (25.17)	224 (28.14)
≥60	5,326 (21.77)	875 (21.00)	928 (19.43)	918 (23.11)	1,506 (22.48)	331 (23.33)	384 (22.83)	384 (21.99)
Region of residence, n (%)								
Urban	12,308 (22.30)	2,146 (22.20)	2,243 (20.39)	2,122 (22.04)	3,426 (22.52)	800 (25.45)	747 (23.96)	824 (24.07)
Rural	3,601 (23.87)	763 (20.76)	634 (20.99)	570 (25.29)	930 (26.39)	206 (27.91)	234 (25.63)	264 (27.50)
Level of education, n (%)								
Middle school or lower education	5,518 (24.48)	1,289 (24.18)	1,129 (22.37)	967 (25.88)	1,346 (25.29)	246 (25.84)	277 (25.18)	264 (25.05)
College or higher education	10,391 (21.77)	1,620 (20.22)	1,748 (19.47)	1,725 (21.18)	3,010 (22.44)	760 (25.95)	704 (24.02)	824 (24.74)
Household income level, n (%)								
Low	3,142 (22.99)	656 (22.84)	548 (19.94)	533 (24.52)	858 (24.02)	173 (26.41)	186 (23.37)	188 (22.17)
Low-medium	4,171 (23.78)	777 (23.43)	789 (21.80)	728 (23.90)	1,128 (24.42)	239 (26.03)	239 (24.95)	271 (25.54)
Medium-high	4,397 (22.97)	745 (20.87)	819 (21.34)	740 (22.62)	1,203 (23.90)	293 (26.40)	285 (25.72)	312 (25.81)
High	4,199 (21.06)	731 (20.40)	721 (18.88)	691 (20.39)	1,167 (21.18)	301 (25.13)	271 (23.18)	317 (25.04)
Level of stress, n (%)								

High stress level	4,527 (24.83)	885 (23.71)	797 (22.14)	703 (24.67)	1,294 (26.28)	307 (29.07)	250 (24.93)	291 (27.38)
Low stress level	11,382 (21.87)	2,024 (21.07)	2,080 (19.96)	1,989 (22.02)	3,062 (22.17)	699 (24.74)	731 (24.14)	797 (24.00)
Drinking status, n (%)								
Non-drinker	4,861 (23.90)	944 (23.66)	873 (21.58)	840 (24.80)	1,293 (25.07)	291 (24.94)	311 (23.14)	309 (24.80)
Drinks more than once per month	11,048 (22.12)	1,965 (21.01)	2,004 (20.09)	1,852 (21.80)	3,063 (22.56)	715 (26.34)	670 (24.93)	779 (24.82)
Smoking status, n (%)								
Smoker or ex-smoker	6,671 (24.44)	1,197 (22.68)	1,164 (21.18)	1,116 (24.73)	1,846 (25.56)	452 (29.95)	403 (26.15)	493 (28.30)
Non-smoker	9,238 (21.50)	1,712 (21.23)	1,713 (20.09)	1,576 (21.39)	2,510 (21.80)	554 (23.36)	578 (23.21)	595 (22.52)

Abbreviations: CI, confidence interval; KNHANES, Korea National Health and Nutrition Examination Survey; SLD, steatotic liver disease.

Table S4. Comparative analysis of MASLD and associated factors, before and during the COVID-19 pandemic, stratified by household income level (weighted % [95% CI]).

Variables	Household income level	Overall (2005–2022)		Before pandemic (2005–2019)		During pandemic (2020–2022)		Ratio of aORs (95% CI) during the pandemic compared to before the pandemic (reference)	
		aOR (95% CI)	P-value	aOR (95% CI)	P-value	aOR (95% CI)	P-value	Weighted ratio of aOR (95% CI)	P-value
Overall	High	1.00 (ref)		1.00 (ref)		1.00 (ref)		1.00 (ref)	
	Medium-high	**1.15 (1.08 to 1.23)**	**<0.001**	**1.19 (1.10 to 1.28)**	**<0.001**	1.08 (0.95 to 1.23)	0.215	0.91 (0.79 to 1.06)	0.216
	Low-medium	**1.18 (1.10 to 1.26)**	**<0.001**	**1.23 (1.14 to 1.32)**	**<0.001**	1.11 (0.97 to 1.28)	0.113	0.91 (0.77 to 1.06)	0.233
	Low	**1.18 (1.10 to 1.27)**	**<0.001**	**1.27 (1.17 to 1.38)**	**<0.001**	0.98 (0.84 to 1.15)	0.680	**0.77 (0.65 to 0.92)**	**0.004**
Sex									
Male	High	1.00 (ref)		1.00 (ref)		1.00 (ref)		1.00 (ref)	
	Medium-high	0.99 (0.92 to 1.07)	0.821	1.02 (0.94 to 1.12)	0.606	0.94 (0.80 to 1.10)	0.450	0.92 (0.76 to 1.10)	0.361
	Low-medium	**0.85 (0.78 to 0.92)**	**<0.001**	**0.87 (0.79 to 0.96)**	**0.007**	0.84 (0.71 to 1.00)	0.050	0.96 (0.79 to 1.17)	0.719
	Low	**0.67 (0.60 to 0.74)**	**<0.001**	**0.71 (0.63 to 0.80)**	**<0.001**	**0.58 (0.47 to 0.72)**	**<0.001**	0.82 (0.64 to 1.04)	0.106
Female	High	1.00 (ref)		1.00 (ref)		1.00 (ref)		1.00 (ref)	
	Medium-high	**1.22 (1.12 to 1.32)**	**<0.001**	**1.26 (1.15 to 1.38)**	**<0.001**	1.10 (0.91 to 1.33)	0.339	0.87 (0.70 to 1.07)	0.195
	Low-medium	**1.53 (1.41 to 1.67)**	**<0.001**	**1.59 (1.45 to 1.74)**	**<0.001**	**1.42 (1.18 to 1.72)**	**<0.001**	0.90 (0.73 to 1.11)	0.309
	Low	**1.96 (1.80 to 2.14)**	**<0.001**	**2.09 (1.90 to 2.30)**	**<0.001**	**1.64 (1.34 to 2.01)**	**<0.001**	**0.79 (0.63 to 0.98)**	**0.033**
Age (years)									
20-29	High	1.00 (ref)		1.00 (ref)		1.00 (ref)		1.00 (ref)	
	Medium-high	1.05 (0.88 to 1.25)	0.582	1.03 (0.84 to 1.27)	0.750	1.13 (0.80 to 1.60)	0.490	1.09 (0.73 to 1.63)	0.661
	Low-medium	1.10 (0.92 to 1.33)	0.292	1.07 (0.87 to 1.33)	0.517	1.39 (0.95 to 2.01)	0.086	1.29 (0.84 to 1.98)	0.240
	Low	1.09 (0.86 to 1.38)	0.476	0.96 (0.72 to 1.27)	0.769	1.52 (0.96 to 2.39)	0.071	1.58 (0.93 to 2.70)	0.091

30–39	High	1.00 (ref)		1.00 (ref)		1.00 (ref)		1.00 (ref)	
	Medium-high	**1.23 (1.08 to 1.40)**	**0.002**	**1.23 (1.06 to 1.43)**	**0.006**	**1.34 (1.01 to 1.76)**	**0.040**	1.09 (0.80 to 1.49)	0.601
	Low-medium	**1.33 (1.15 to 1.53)**	**<0.001**	**1.43 (1.22 to 1.67)**	**<0.001**	1.22 (0.87 to 1.72)	0.253	0.86 (0.59 to 1.25)	0.418
	Low	**1.29 (1.03 to 1.63)**	**0.029**	**1.46 (1.14 to 1.88)**	**0.003**	0.90 (0.49 to 1.66)	0.743	0.62 (0.32 to 1.19)	0.150
40–49	High	1.00 (ref)		1.00 (ref)		1.00 (ref)		1.00 (ref)	
	Medium-high	**1.25 (1.11 to 1.40)**	**<0.001**	**1.31 (1.15 to 1.50)**	**<0.001**	1.03 (0.80 to 1.32)	0.842	0.78 (0.59 to 1.03)	0.085
	Low-medium	**1.23 (1.08 to 1.40)**	**0.001**	**1.28 (1.11 to 1.48)**	**0.001**	1.12 (0.86 to 1.45)	0.419	0.87 (0.65 to 1.18)	0.374
	Low	**1.27 (1.05 to 1.53)**	**0.014**	**1.23 (1.00 to 1.52)**	**0.050**	**1.61 (1.04 to 2.48)**	**0.031**	1.30 (0.81 to 2.10)	0.278
50–59	High	1.00 (ref)		1.00 (ref)		1.00 (ref)		1.00 (ref)	
	Medium-high	1.11 (0.99 to 1.25)	0.084	**1.22 (1.06 to 1.39)**	**0.004**	0.88 (0.68 to 1.13)	0.313	**0.72 (0.54 to 0.96)**	**0.027**
	Low-medium	**1.19 (1.05 to 1.35)**	**0.006**	**1.20 (1.04 to 1.38)**	**0.013**	**1.36 (1.04 to 1.79)**	**0.026**	1.14 (0.84 to 1.55)	0.403
	Low	**1.33 (1.14 to 1.56)**	**<0.001**	**1.39 (1.18 to 1.65)**	**<0.001**	1.30 (0.90 to 1.88)	0.159	0.93 (0.62 to 1.40)	0.737
≥60	High	1.00 (ref)		1.00 (ref)		1.00 (ref)		1.00 (ref)	
	Medium-high	1.01 (0.89 to 1.15)	0.869	1.05 (0.90 to 1.23)	0.549	0.95 (0.74 to 1.21)	0.685	0.91 (0.68 to 1.21)	0.507
	Low-medium	0.93 (0.82 to 1.05)	0.221	0.97 (0.84 to 1.11)	0.621	0.87 (0.69 to 1.10)	0.242	0.90 (0.69 to 1.18)	0.464
	Low	0.91 (0.81 to 1.02)	0.118	1.02 (0.89 to 1.17)	0.787	**0.70 (0.56 to 0.88)**	**0.002**	**0.69 (0.53 to 0.90)**	**0.006**
Region of residence									
Urban	High	1.00 (ref)		1.00 (ref)		1.00 (ref)		1.00 (ref)	
	Medium-high	1.05 (0.99 to 1.12)	0.127	**1.09 (1.01 to 1.17)**	**0.026**	0.98 (0.86 to 1.12)	0.776	0.90 (0.77 to 1.05)	0.182
	Low-medium	**1.07 (1.00 to 1.15)**	**0.041**	**1.11 (1.03 to 1.20)**	**0.006**	1.03 (0.89 to 1.19)	0.695	0.92 (0.79 to 1.09)	0.337
	Low	**1.15 (1.07 to 1.24)**	**<0.001**	**1.25 (1.15 to 1.36)**	**<0.001**	0.93 (0.79 to 1.10)	0.388	**0.74 (0.62 to 0.89)**	**0.002**
Rural	High	1.00 (ref)		1.00 (ref)		1.00 (ref)		1.00 (ref)	
	Medium-high	1.12 (0.98 to 1.29)	0.110	**1.19 (1.01 to 1.41)**	**0.043**	0.91 (0.71 to 1.18)	0.482	0.77 (0.57 to 1.04)	0.087
	Low-medium	1.05 (0.91 to 1.21)	0.495	1.12 (0.95 to 1.33)	0.184	0.87 (0.69 to 1.09)	0.221	0.77 (0.58 to 1.03)	0.075
	Low	0.97 (0.84 to 1.13)	0.703	1.04 (0.88 to 1.24)	0.629	0.75 (0.56 to 1.02)	0.063	0.72 (0.52 to 1.01)	0.060

Level of education									
Middle school or lower education	High	1.00 (ref)		1.00 (ref)		1.00 (ref)		1.00 (ref)	
	Medium-high	**1.18 (1.03 to 1.34)**	**0.018**	**1.22 (1.05 to 1.41)**	**0.010**	0.97 (0.71 to 1.33)	0.864	0.80 (0.57 to 1.13)	0.205
	Low-medium	**1.25 (1.10 to 1.42)**	**<0.001**	**1.29 (1.12 to 1.49)**	**<0.001**	1.01 (0.75 to 1.36)	0.931	0.78 (0.57 to 1.09)	0.143
	Low	**1.32 (1.16 to 1.49)**	**<0.001**	**1.37 (1.20 to 1.57)**	**<0.001**	1.05 (0.79 to 1.39)	0.748	0.76 (0.56 to 1.05)	0.095
College or higher education	High	1.00 (ref)		1.00 (ref)		1.00 (ref)		1.00 (ref)	
	Medium-high	**1.11 (1.04 to 1.19)**	**0.002**	**1.14 (1.05 to 1.23)**	**<0.001**	1.06 (0.93 to 1.21)	0.383	0.93 (0.80 to 1.09)	0.373
	Low-medium	**1.14 (1.06 to 1.22)**	**<0.001**	**1.16 (1.07 to 1.27)**	**<0.001**	1.15 (0.99 to 1.33)	0.077	0.98 (0.83 to 1.17)	0.860
	Low	1.02 (0.92 to 1.13)	0.727	1.06 (0.94 to 1.19)	0.340	0.94 (0.77 to 1.15)	0.572	0.89 (0.71 to 1.12)	0.335
Stress level									
High stress level	High	1.00 (ref)		1.00 (ref)		1.00 (ref)		1.00 (ref)	
	Medium-high	**1.17 (1.05 to 1.31)**	**0.005**	**1.15 (1.02 to 1.30)**	**0.027**	1.25 (0.99 to 1.59)	0.063	1.09 (0.84 to 1.43)	0.522
	Low-medium	1.12 (1.00 to 1.26)	0.057	**1.16 (1.02 to 1.32)**	**0.027**	1.06 (0.82 to 1.38)	0.648	0.92 (0.69 to 1.23)	0.568
	Low	**1.15 (1.01 to 1.30)**	**0.032**	**1.16 (1.01 to 1.34)**	**0.034**	1.20 (0.90 to 1.60)	0.219	1.03 (0.75 to 1.42)	0.860
Low stress level	High	1.00 (ref)		1.00 (ref)		1.00 (ref)		1.00 (ref)	
	Medium-high	**1.09 (1.02 to 1.17)**	**0.015**	**1.16 (1.07 to 1.26)**	**<0.001**	0.94 (0.81 to 1.08)	0.381	**0.81 (0.69 to 0.95)**	**0.011**
	Low-medium	**1.14 (1.06 to 1.23)**	**0.000**	**1.19 (1.10 to 1.29)**	**<0.001**	1.08 (0.93 to 1.25)	0.317	0.91 (0.76 to 1.08)	0.262
	Low	1.07 (0.99 to 1.16)	0.100	**1.19 (1.09 to 1.31)**	**<0.001**	**0.79 (0.67 to 0.94)**	**0.007**	**0.66 (0.55 to 0.80)**	**<0.001**
Drinking status									
Non-drinker	High	1.00 (ref)		1.00 (ref)		1.00 (ref)		1.00 (ref)	
	Medium-high	**1.21 (1.08 to 1.36)**	**0.001**	**1.26 (1.11 to 1.43)**	**<0.001**	1.09 (0.85 to 1.40)	0.502	0.87 (0.65 to 1.15)	0.316
	Low-medium	**1.33 (1.19 to 1.49)**	**<0.001**	**1.32 (1.16 to 1.51)**	**<0.001**	**1.35 (1.07 to 1.70)**	**0.010**	1.02 (0.78 to 1.33)	0.884
	Low	**1.42 (1.27 to 1.58)**	**<0.001**	**1.55 (1.37 to 1.75)**	**<0.001**	1.06 (0.84 to 1.35)	0.614	**0.69 (0.52 to 0.90)**	**0.006**
	High	1.00 (ref)							
	Medium-high	**1.09 (1.02 to 1.17)**	**0.012**	**1.13 (1.04 to 1.22)**	**0.003**	1.02 (0.88 to 1.17)	0.812	0.90 (0.77 to 1.06)	0.201

Drinks more than once per month	Low-medium	**1.08 (1.01 to 1.17)**	**0.027**	**1.14 (1.05 to 1.24)**	**0.002**	1.03 (0.88 to 1.20)	0.731	0.90 (0.76 to 1.07)	0.237
	Low	0.94 (0.86 to 1.03)	0.188	0.99 (0.90 to 1.10)	0.846	0.88 (0.73 to 1.06)	0.173	0.89 (0.72 to 1.10)	0.266
Smoking status									
Non-smoker	High	1.00 (ref)		1.00 (ref)		1.00 (ref)		1.00 (ref)	
	Medium-high	**1.10 (1.01 to 1.18)**	**0.021**	**1.14 (1.05 to 1.25)**	**0.003**	0.97 (0.83 to 1.14)	0.743	0.85 (0.71 to 1.02)	0.079
	Low-medium	**1.28 (1.19 to 1.39)**	**<0.001**	**1.33 (1.22 to 1.46)**	**<0.001**	**1.20 (1.01 to 1.41)**	**0.036**	0.90 (0.74 to 1.08)	0.258
	Low	**1.61 (1.48 to 1.75)**	**<0.001**	**1.72 (1.57 to 1.89)**	**<0.001**	**1.34 (1.11 to 1.63)**	**0.003**	**0.78 (0.63 to 0.97)**	**0.023**
Smoker or ex-smoker	High	1.00 (ref)		1.00 (ref)		1.00 (ref)		1.00 (ref)	
	Medium-high	1.04 (0.96 to 1.14)	0.353	1.07 (0.97 to 1.19)	0.194	1.02 (0.85 to 1.21)	0.866	0.95 (0.77 to 1.16)	0.613
	Low-medium	**0.88 (0.80 to 0.97)**	**0.009**	0.92 (0.83 to 1.02)	0.114	0.86 (0.70 to 1.05)	0.128	0.93 (0.75 to 1.17)	0.548
	Low	**0.67 (0.60 to 0.75)**	**<0.001**	**0.73 (0.65 to 0.83)**	**<0.001**	**0.56 (0.44 to 0.70)**	**<0.001**	**0.76 (0.59 to 0.98)**	**0.037**

Abbreviations: aOR, adjusted odds ratio; CI, confidence interval; KNHANES, Korea National Health and Nutrition Examination Survey; MASLD, metabolic dysfunction–associated steatotic liver disease.

The values in bold font represent a significant variance (p<0.05).
